# SPiP: Splicing Prediction Pipeline, a machine learning tool for massive detection of exonic and intronic variant effects on mRNA splicing

**DOI:** 10.1002/humu.24491

**Published:** 2022-11-20

**Authors:** Raphaël Leman, Béatrice Parfait, Dominique Vidaud, Emmanuelle Girodon, Laurence Pacot, Gérald Le Gac, Chandran Ka, Claude Ferec, Yann Fichou, Céline Quesnelle, Camille Aucouturier, Etienne Muller, Dominique Vaur, Laurent Castera, Flavie Boulouard, Agathe Ricou, Hélène Tubeuf, Omar Soukarieh, Pascaline Gaildrat, Florence Riant, Marine Guillaud‐Bataille, Sandrine M. Caputo, Virginie Caux‐Moncoutier, Nadia Boutry‐Kryza, Françoise Bonnet‐Dorion, Ines Schultz, Maria Rossing, Olivier Quenez, Louis Goldenberg, Valentin Harter, Michael T. Parsons, Amanda B. Spurdle, Thierry Frébourg, Alexandra Martins, Claude Houdayer, Sophie Krieger

**Affiliations:** ^1^ Laboratoire de Biologie et Génétique du Cancer Centre François Baclesse Caen France; ^2^ Inserm U1245, UNIROUEN, FHU‐G4 génomique Normandie Université Rouen France; ^3^ UNICAEN Normandie Université Caen France; ^4^ Service de Génétique et Biologie Moléculaires, APHP, HUPC Hôpital Cochin Paris France; ^5^ Inserm UMR1078, Genetics, Functional Genomics and Biotechnology Université de Bretagne Occidentale Brest France; ^6^ Integrative Biosoftware Rouen France; ^7^ Laboratoire de Génétique, AP‐HP GH Saint‐Louis‐Lariboisière‐Fernand Widal Paris France; ^8^ Département de Biopathologie, Gustave Roussy Université Paris‐Saclay Villejuif France; ^9^ Department of Genetics, Institut Curie Paris Sciences Lettres Research University Paris France; ^10^ Unité Mixte de Génétique Constitutionnelle des Cancers Fréquents Hospices Civils de Lyon Lyon France; ^11^ Departement de Biopathologie Unité de Génétique Constitutionnelle Institut Bergonie—INSERM U1218 Bordeaux France; ^12^ Laboratoire d'Oncogénétique Centre Paul Strauss Strasbourg France; ^13^ Centre for Genomic Medicine, Rigshospitalet University of Copenhagen Copenhagen Denmark; ^14^ Department of Biostatistics Baclesse Unicancer Center Caen France; ^15^ Department of Genetics and Computational Biology QIMR Berghofer Medical Research Institute Herston Queensland Australia; ^16^ Department of genetics Rouen University Hospital Rouen France

**Keywords:** machine learning, RNA, sequence variants, SPiP, splicing predictions

## Abstract

Modeling splicing is essential for tackling the challenge of variant interpretation as each nucleotide variation can be pathogenic by affecting pre‐mRNA splicing via disruption/creation of splicing motifs such as 5′/3′ splice sites, branch sites, or splicing regulatory elements. Unfortunately, most in silico tools focus on a specific type of splicing motif, which is why we developed the Splicing Prediction Pipeline (SPiP) to perform, in one single bioinformatic analysis based on a machine learning approach, a comprehensive assessment of the variant effect on different splicing motifs. We gathered a curated set of 4616 variants scattered all along the sequence of 227 genes, with their corresponding splicing studies. The Bayesian analysis provided us with the number of control variants, that is, variants without impact on splicing, to mimic the deluge of variants from high‐throughput sequencing data. Results show that SPiP can deal with the diversity of splicing alterations, with 83.13% sensitivity and 99% specificity to detect spliceogenic variants. Overall performance as measured by area under the receiving operator curve was 0.986, better than SpliceAI and SQUIRLS (0.965 and 0.766) for the same data set. SPiP lends itself to a unique suite for comprehensive prediction of spliceogenicity in the genomic medicine era. SPiP is available at: https://sourceforge.net/projects/splicing-prediction-pipeline/

## 1 INTRODUCTION

Modeling RNA splicing is an essential aspect of variant interpretation. Splicing alterations are implicated in a large variety of disease phenotypes and are presumably the most frequent alterations involved in hereditary diseases (López‐Bigas et al., 2005; Wang & Cooper, 2007). The reason is that each nucleotide variation, regardless of its location (exon/intron), can potentially impact splicing. Recent data showed that close to 4% of ExAC variants lead to a splice alteration (Cheung et al., 2019), making their detection mandatory for the genetic diagnosis of hereditary and somatic diseases and more broadly for genomic medicine. Splice alterations are highly diverse in nature, for example, single‐ or multiexon skipping, splice site shifts (i.e., use of new splice sites resulting in a partial exonic deletion or a partial intronic retention), intronic exonization with the creation of a pseudo‐exon, and total intronic retention (Supporting Information: Figure S1) (Wimmer et al., 2007). For brevity, we use the term spliceogenic variant to refer to DNA variants that result in splice alterations whatever the nature of alterations. The diversity of splicing alterations results from the disruption and/or creation of one or more splicing motifs. Pre‐mRNA splicing indeed requires a number of *cis*‐acting motifs, the splice donor site (5′ss), the splice acceptor site (3′ss), the branch point (BP), the polypyrimidine tract (PPT) located between the BP and the 3′ss, and auxiliary motifs called splicing regulatory elements located close to the 5′/3′ splice sites (Dominguez et al., 2018) (Supporting Information: Figure S2). Unfortunately, most in silico prediction tools are dedicated to assessing the variant effect on specific splicing motifs. Few tools, such as SpliceAI or SQUIRLS, take into account the full set of splicing motifs (Danis et al., 2021; Jaganathan et al., 2019). While, to date, SpliceAI has shown the best performance compared to other tools, it was trained on wild‐type sequences and not on the splicing impact of variants.

To fill this gap, we developed Splicing Prediction Pipeline (SPiP), as an application suite running a cascade of optimal and complementary bioinformatics tools to prioritize RNA in vitro studies irrespective of variant position (exonic, intronic, or deep intronic). These tools were gathered within a unique model by a machine learning approach based on the random forest to get a global score of splicing alteration. We collected a curated set of 4616 variants detected in 227 genes involved in 161 clinical signs and syndromes, with their corresponding splicing studies. These variants occurred in a wide spatial range relative to the splice sites, up to 36 kb from the closest natural splice site. SPiP detected all classes of splice alterations (exon skipping, splice site shift, pseudo‐exon, and intronic retention) in this large variant collection, reaching a high value of sensitivity and specificity.

## 2 MATERIALS AND METHODS

### 2.1 Definition of splicing defects

Four classes of splicing defects were defined (Supporting Information: Figure S1): (i) exon skipping, for example, single‐ and multiple‐exon cassette skipping; (ii) splice site shift; (iii) creation of pseudo‐exons deeply within introns; and (iv) full intronic retention. Splice site shifts result from four mechanisms (Supporting Information: Figure S3): (A) a variant disrupts a natural splice site and concomitantly causes the use of a new splice site, instead of exon skipping (shift of natural splice site), (B) a variant creates a new splice site in a nucleotide context without any predicted splice site (de novo splice site), (C) a variant creates a new splice site in the context of a predicted but unused splice site (cryptic splice site), and (D) a variant creates a de novo splice site and activates a cryptic splice site. We also studied the distance between the natural splice site and the new splice site and the distance between the variant and the new splice site. Both partial and total transcript alterations were considered as a unique splicing alteration; quantitative aspects were not considered.

### 2.2 Variant collection with RNA studies

This curated set was made of 4616 variants (1924 spliceogenic variants) from 227 genes with results from corresponding RNA in vitro studies (4114 published and 502 unpublished; Supporting Information: Figure S4 and Table S1). The published data were obtained from: reports of variants with their RNA in vitro studies (*N* = 2816) (Anna & Monika, 2018; Leman et al., 2018; Lewandowska, 2013; Tubeuf et al., 2020; Woolfe et al., 2010), the DBASS5 and DBASS3 databases (*N* = 111) (Buratti et al., 2007; Vořechovský, 2006), literature data collected by the ENIGMA (Evidence‐based Network for the Interpretation of Germline Mutant Alleles) (Spurdle et al., 2012) consortium (*N* = 448), and *Service de Génétique et Biologie Moléculaires*, Cochin hospital (*N* = 739). We aggregated these data until 2020. Data were manually curated for genomic variant and for the splicing alteration by the perusal of the original articles. The 502 unpublished variants were studied for diagnostic purposes by the French network of diagnostic laboratories, Unicancer Genetic Group (UGG, http://www.unicancer.fr/en/cancer-and-genetic-group), and Association Nationale des Praticiens de Génétique Moléculaire (ANPGM, https://anpgm.fr/): (i) the *Service de Génétique et Biologie Moléculaires*, Cochin Hospital (*N* = 230), (ii) the UGG (*N* = 167), (iii) the Inserm U1078 Laboratory (*N* = 63), (iv) the Inserm U1245 Laboratory (*N* = 22), (v) the laboratory of genetics of the *Saint‐Louis‐Lariboisière‐Fernand Widal* Hospital (*N* = 17), and (vi) the Center of genomics of the University of Copenhagen (*N* = 3). Briefly, protocols for transcript analyses included RNA extracted from whole blood collected on PAXgene^TM^ tubes and/or lymphoblastoid cell lines treated or untreated with the NMD inhibitor puromycin, and were based on RT‐PCR analyses including Sanger sequencing of RT‐PCR products and/or on cell‐based minigene splicing assays (Callebaut et al., 2014; Gaildrat et al., 2010; Houdayer et al., 2012; Riant et al., 2013; Sabbagh et al., 2013; Steffensen et al., 2014).

### 2.3 Building comprehensive data collection

High‐throughput sequencing identifies a large number of variants, of which the proportion of spliceogenic variants has not been accurately estimated. From the collection of variants with RNA studies, we estimated the probability to get a spliceogenic variant (P(S)) within 227 genes of the variant collection with RNA studies. A Bayesian approach was used to compute this probability (see Supporting Information: Figure S5 and Methods section for details). We found a proportion P(S) of 2.08% of spliceogenic variants. In other words, to obtain 1924 spliceogenic variants, we would have had, in theory, to study approximately 100,000 variants. The latest and largest high‐output splicing assay was limited to 27,000 variants (Cheung et al., 2019) and explored only exon‐skipping events from variants in or close to exonic regions. Thus, to develop SPiP under the condition where most variants detected do not impact splicing, we had to use a collection of “control variants,” presumed to have no impact on disease risk via splicing, to be in line with this probability P(S). These control variants were common variants (minor allele frequency >5%) within the 227 genes for which variant‐related assay data was available. Indeed, among the 227 genes, 82% of them (186/227) were clearly linked to clinical signs and syndromes (Supporting Information: Table S2) (Maiella et al., 2013). Common variants (minor allele frequency >5%), for at least 82% out of 227 genes, do not impact the gene function. Thus, we made the hypothesis that common frequent variants have negligible impact on splicing.

In all, 95,000 control variants were added to the 4616 variants for a total collection of 99,616 variants. This novel collection was used to develop SPiP and used for comparison with other tools.

### 2.4 Selection of bioinformatic tools for analysis of consensus splice sites, BP, PPT, and exonic splicing regulators (ESRs)

**Figure 1:**
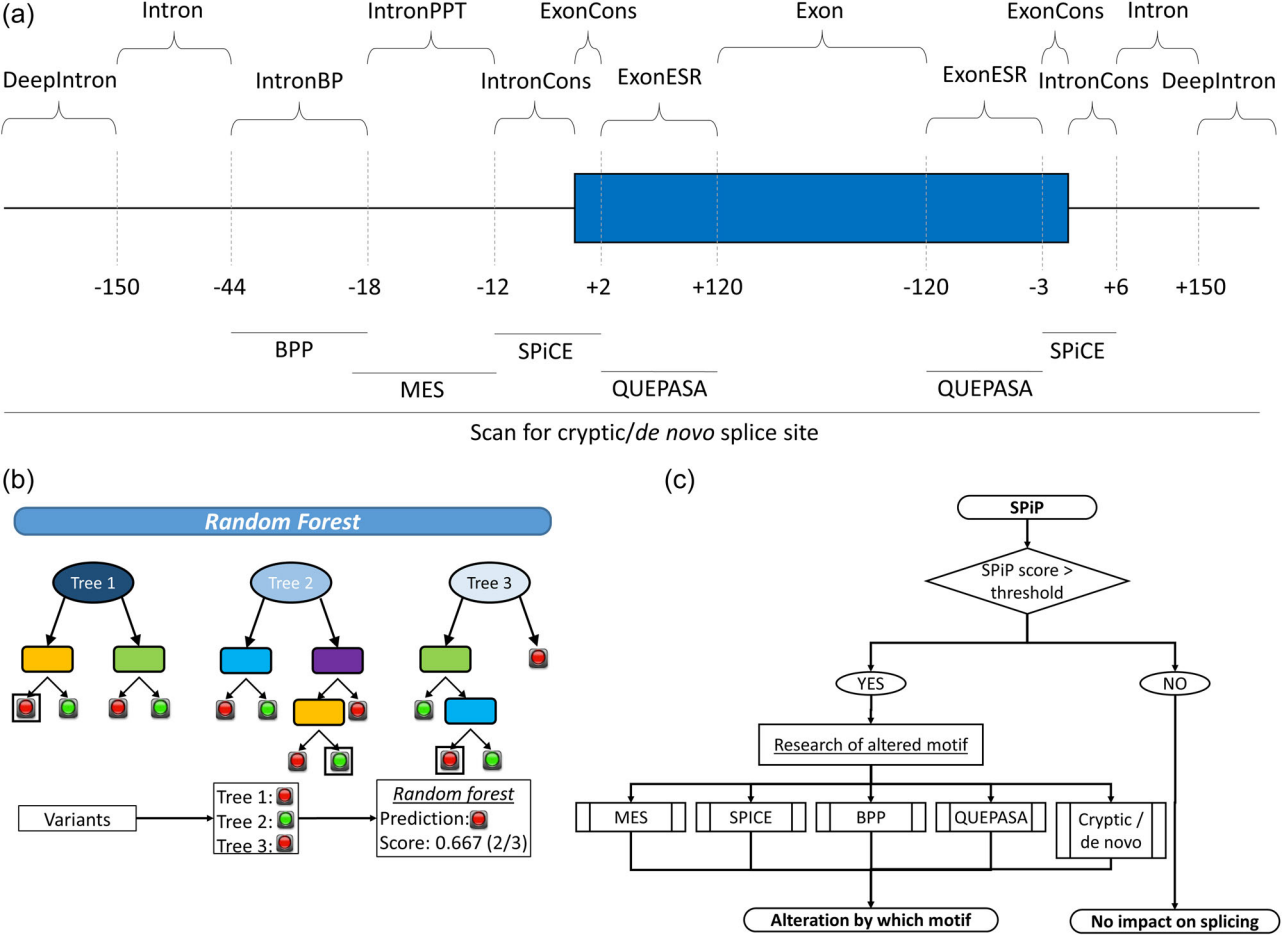
SPiP workflow. (a) The position of splicing motifs and their corresponding tool within the gene sequence. (b) Principle of random forest. The model generates several decision trees to classify variants according to the predictors, then the final outcome is the proportion of trees predicting an alteration. (c) Annotation pipeline of SPiP. In addition to the random forest predictions, SPiP displays which motifs are probably altered based on predictions of tools implemented in SPiP (Supporting Information: Figure S7). BPP, Branch Point Predictor; IntronBP, branch point region; IntronCons, intronic consensus splice site (either 3′ss or 5′ss); IntronPPT, polypyrimidine tract; ExonCons, exonic consensus splice site; MES, MaxEntScan; QUEPASA, Quantifying Extensive Phenotypic Arrays from Sequence Arrays; SPiCE, Splicing Prediction in Consensus Element; SPiP, Splicing Prediction Pipeline.

SPiP is built on a cascade of complementary tools addressing the impact of the variant on different splicing motifs. The tools were selected to fulfill the following requirements: (i) availability at the time of initiating this work, (ii) possibility to implement in a pipeline for high‐throughput, batch analyses (i.e., exclusion of web‐based tools), (iii) previously published studies have reported on their performance (named benchmark studies hereinafter), and (iv) free to use. Hence, we considered 15 tools evaluated by six benchmark studies (Grodecká, Buratti, et al. 2017; Grodecká, Hujová, et al., 2017; Houdayer et al., 2012; Leman et al., 2018, 2020; Soukarieh et al., 2016), described in Table 1. According to these studies, splicing prediction in consensus element, MaxEntScan (MES), BP Prediction, and Quantifying Extensive Phenotypic Arrays from Sequence Arrays were found to be optimal and selected for consensus splice sites, PPT, BP, and ESRs, respectively (Figure 1a).

**Table 1:** Bioinformatics tools considered

Tool	Method	Cons 5′/3′ss	PPT tract	Branch point	ESRs	Benchmark studies
SpliceSite Finder (SSF) (Shapiro & Senapathy, 1987)	PWM Trained on immunoglobulin consensus sequences Not available but easily implementable	+	−	−	−	Houdayer et al. (2012), Leman et al. (2018)
MaxEntScan (MES) (Yeo & Burge, 2004)	Maximum entropy Trained on RNA‐sequencing data http://hollywood.mit.edu/burgelab/maxent/Xmaxentscan_scoreseq.html	+	+	−	−	Houdayer et al. (2012), Leman et al. (2018)
Human Splicing Finder (HSF) (Desmet et al., 2009)	PWM Trained on conserved sequences from the Ensembl transcripts http://www.umd.be/HSF3/	+	−	+	+	Houdayer et al. (2012), Leman et al. (2018, 2020)
GeneSplicer (GS) (Pertea et al., 2001)	Decision tree (maximal dependence decomposition) *Arabidopsis thaliana* and human splice sites https://www.cbcb.umd.edu/software/GeneSplicer/gene_spl.shtml	+	+	−	‐	Houdayer et al. (2012), Leman et al. (2018)
Neural Network Splice (NNS) (Reese et al., 1997)	Neural network Trained on human splice sites https://www.fruitfly.org/seq_tools/splice.html	+	+	−	−	Houdayer et al. (2012), Leman et al. (2018)
SVM‐BPfinder (Corvelo et al., 2010)	Support vector machine combining BP predictions and PPT features Trained on conserved sequences from seven species (including humans) http://regulatorygenomics.upf.edu/Software/SVM_BP/	−	−	+	−	Leman et al. (2020)
Exon‐Skipping (EX‐SKIP) (Raponi et al., 2011)	Ratio ESE/ESS Trained on exonic variants of *BRCA1* https://ex-skip.img.cas.cz/	−	−		+	Grodecká, Buratti et al. (2017), Grodecká, Hujová et al. (2017), Soukarieh et al. (2016)
Quantifying Extensive Phenotypic Arrays from Sequence Arrays (QUEPASA) (Di Giacomo et al., 2013)	Ratio score ESE/ESS Trained on exonic variants of *BRCA2* Not available but easily implementable	−	−	−	+	Grodecká, Buratti et al. (2017), Grodecká et al. (2017), Soukarieh et al. (2016)
HEXplorer (∆HZ_EI_) (Erkelenz et al., 2014)	*Z*‐score ESR Trained on exonic variants https://www2.hhu.de/rna/html/hexplorer_score.php	‐	‐	‐	+	Grodecká, Buratti et al. (2017), Grodecká et al. (2017), Soukarieh et al. (2016)
Splicing‐based analysis of variants (SPANR or SPIDEX) (Xiong et al., 2015)	Deep learning based on a computational system Trained on RNA‐sequencing data http://tools.genes.toronto.edu/	+	+	+	+	Grodecká, Buratti, et al. (2017), Grodecká, Hujová et al. (2017), Soukarieh et al., 2016)
Branch Point Prediction (BPP) (Zhang et al., 2017)	Mixture model combining BP predictions and PPT features Trained on conserved sequences from human introns https://github.com/zhqingit/BPP	−	−	+	−	Leman et al. (2020)
Branchpointer (Signal et al., 2018)	Machine learning based on primary and secondary structures of RNA Trained on high‐confidence BPs (Mercer et al., 2015) https://doi.org/10.18129/B9.bioc.branchpointer	−	−	+	−	Leman et al. (2020)
Splicing prediction in consensus element (SPiCE) (Leman et al., 2018)	Metascore (SSF + MES) Trained on consensus variants https://sourceforge.net/projects/spicev2-1/	+	−	−	−	Leman et al. (2018)
LSTM Branch point Retriever (LaBranchoR) (Paggi & Bejerano, 2018)	Deep learning based on bidirectional LSTM Trained on high‐confidence BPs (Mercer et al., 2015) http://bejerano.stanford.edu/labranchor/	−	−	+	−	Leman et al. (2020)
RNA Branch Point Selection (RNABPS) (Nazari et al., 2019)	Deep learning based on dilated convolution and bidirectional LSTM Trained on high‐confidence BPs (Mercer et al., 2015; Pineda & Bradley, 2018) https://home.jbnu.ac.kr/NSCL/rnabps.htm	−	−	+	−	Leman et al. (2020)

*Note*: All of these tools were free to use except HSF, but HSF's authors proposed a web page to score variants without charge.

Abbreviations: LSTM, long short‐term memory network; PPT, polypyrimidine tract between the 3′ consensus splice site and the branch point region (−13 to −17); PWM, position weight matrix.

### 2.5 Definition of a new metascore to predict splice site creation or reinforcement (de novo/cryptic)

Given the lack of published benchmark studies on splice site creation or reinforcement, we developed a new model to detect the use of de novo/cryptic splice sites regardless of the position of the variant on the genomic sequence. This new model provides a metascore based on a logistic regression that includes the scores of consensus splice sites and ESR motifs. Consensus splice sites were scored using MES and the SSF‐like position weight matrix as we previously found this combination to be optimal (Jian et al., 2014; Leman et al., 2018). ESR motifs were scored by using ESRseq values obtained by large‐scale minigene‐splicing assays (Ke et al., 2011). The resulting metascores give the probability of splice site use. The model was trained and validated on a large set of true and false splice sites. The true splice sites were defined from Ensembl transcripts (downloaded June 28, 2018) (*N* = 530,931 true splice sites). The false sites were both AG and GT motifs outside true splice sites in the Ensembl transcripts, corresponding to a comprehensive list of random AG/GT (*N* = 202,458,725 negative sites), that is, the ratio of true splice sites to false splice sites was 1:381. Two‐thirds of this data collection was used as a training set and the remaining third as a validation set. Then, we used receive‐operating characteristic (ROC) analysis to compare the performance of each tool (MES, SSF‐like, ESRscore) against our metascore using the evaluation set. The strategy to detect splice site activation from this model is illustrated in Supporting Information: Figure S6. Briefly, we compared the scores of potential splice sites around the variant between wild‐type and variant sequences. Reinforced splice sites and new splice sites displaying the maximal scores were integrated into the SPiP algorithm.

### 2.6 SPiP setting

To predict splicing impact, we used a metascore from a model based on a random forest classifier. R software v3.5.1 with the library randomForest v4.6‐14 were used to build the model (Figure 1b). Briefly, a random forest classifier is a machine learning based on the automatic training of hundreds of decision trees. Each tree uses a random subset of explicative variables and classifies the observation in two or more categories. Then, the random forest model gives a score between 0 and 1 corresponding to the ratio of the number of trees with positive classification to the total number of trees. This score, hereafter called SPiP score, was used to classify a variant regarding its splicing impact. The tested predictors were the different scores for each splicing motif plus the exon/intron sizing and the relative position of variants. We defined the training and validation set of data by randomly splitting our data (*N* = 99,616 variants) into two independent groups with the same number of variants (*N* = 49,808 variants). We chose the optimal predictors based on their importance, that is, their capability to decrease the error rate of the model, and also based on the model performance on the validation set of data. Then, the backward variable selection gave us a final model. We also adjusted the number of predictors sampled for splitting at each node by the same approach. To evaluate the performance of the SPiP score and to define an optimal threshold, we used ROC analyses. The decision‐making threshold was set to get an optimal specificity to decrease the false discovery rate.

In addition to the SPiP score, two additional pieces of information were added to increase the interpretability of SPiP predictions. First, for variants with positive predictions, SPiP displays which motif is probably impacted by the variants (Figure 1c and Supporting Information: Figure S7). Briefly, for each published tool gathered in SPiP, we applied the decision threshold recommended by tool authors, and for the de novo/cryptic model, we applied a threshold to get a specificity of 98%. Second, based on the observed proportion of spliceogenic variants according to the SPiP score, we calculated confidence intervals (CIs). The variants were distributed in different categories according to the score values to have a similar group size between categories. Then, the CI of the proportion of spliceogenic variants was calculated for each category. Regarding negative predictions, the CIs were calculated from the proportion of false negatives among true‐negative variants, split into groups according to the relative position of variants.

The SPiP tool locates the variant within the transcripts described from the RefSeq database with the assembly genome version hg19 and hg38. The input of SPiP requires the reference transcript ID (RefSeq) as well as the ID of the variant in HGVS (Human Genome Variation Society) (den Dunnen et al., 2016) nomenclature (e.g., NM_007294:c.4096+3A>G). SPiP was developed to support Variant Call Format v4.0 or later, or standardized text file formats. SPiP runs an R script to calculate the scores (available at https://sourceforge.net/projects/splicing-prediction-pipeline/) in a standalone version, thus supplemental installation is not necessary. SPiP was also encoded to perform batch analysis on Linux machine, with the possibility to parallelize the calculation (https://github.com/raphaelleman/SPiP).

### 2.7 SPiP versus SpliceAI and SQUIRLS

SPiP was compared to the deep learning tool SpliceAI (Jaganathan et al., 2019), and to another random forest tool SQUIRLS (Danis et al., 2021). SQUIRLS is a recently developed tool similar in design to SPiP, but using nucleotide conservation to predict the impact of a variant. For comparison, we used the docker file available at https://hub.docker.com/r/urpin/squirls, which embedded version 2.0.0 of SQUIRLS. At the time of study initiation, SpliceAI was one of the latest splicing prediction tools and had optimal performance when compared to previously published tools, owing to a 32‐layer deep neural network. This tool (v1.3) was installed on our Linux machine from the GitHub repository: https://github.com/Illumina/SpliceAI. To avoid any bias, the comparison was performed on the total collection of variants (*n* = 99,616). However, SpliceAI could not score 914 variants among them; therefore, the comparison was performed on the remaining 98,702. We evaluated the performance of the SPiP and SpliceAI tools by ROC analyses and Precision‐Recall analyses, with R libraries ROCR and PRROC, respectively. The first analysis compared the sensitivity and specificity for each cutoff. The second compared the sensitivity (recall) and the positive prediction value (precision) for each cutoff. We considered the area under the curve (AUC) values and the sensitivities for fixed values of specificity (95%, 96%, 97%, 98%, and 99%) plus the precision values for fixed sensitivity values (80%, 85%, 90%, and 95%). The comparison was done 100 times, each with a new random validation (*N* = 49,350 variants) and training set (*N* = 49,350 variants) of data. The data were split according to the genomic position of the variant. Intel Xeon CPU E5‐2687W v3 @ 3.10 GHz Processor, 200 GB RAM machine was used to evaluate the run time of the three tools.

We completed the comparison by collecting new variants published from 2020 to 2022 with RNA in vitro studies. We removed variants occurring at the same genomic position of variants in the initial collection of 99,616 variants. This new collection had 426 variants occurring in 71 genes (Supporting Information: Table S3). Sixty‐nine percent (49/71) of these genes were not present in the initial collection of 227 genes. We compared the performance of the three scores by ROC analysis. The sensitivity, specificity, and accuracy were calculated according to an optimal threshold (i.e., a threshold with the optimal value of specificity and sensitivity) for each score.

## 3 RESULTS

### 3.1 Description of splicing alteration

**Figure 2:**
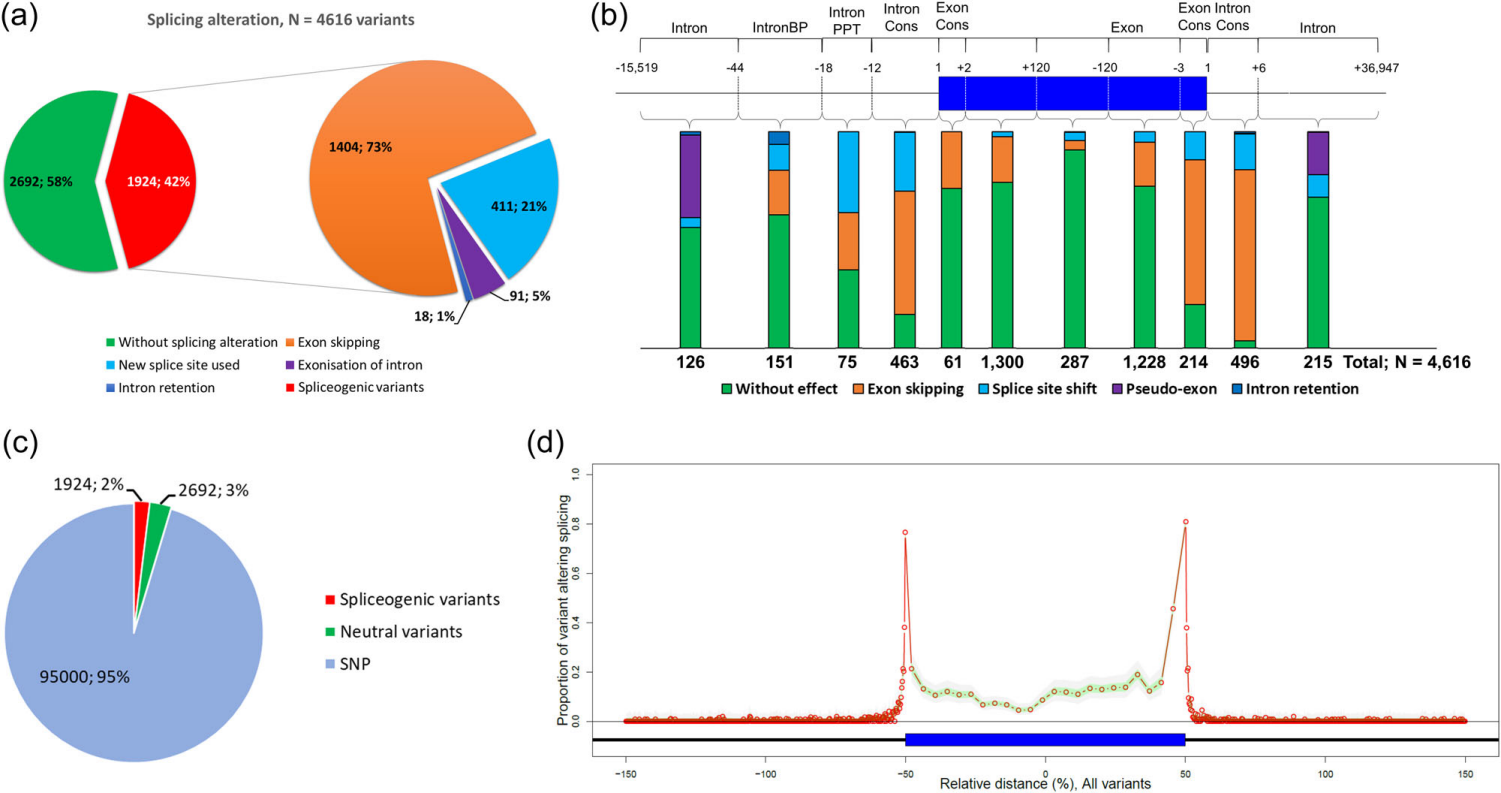
Characteristic of variants used in this study. (a) Distribution of splicing events observed for variants with RNA in vitro studies (*N* = 4616 variants). (b) Variant distribution along the pre‐mRNA molecule with their impact on splicing (*N* = 4616 variants). (c) Variant distribution, including “control variants” (SNPs with frequency >5%) (*N* = 99,616 variants). (d) Proportion of variants that impacts splicing all along the pre‐mRNA molecule (*N* = 99,616 variants). SNP, single‐nucleotide polymorphism.

The total set of 4616 distinct variants from 227 genes included 1924 (41.68%) proven spliceogenic variants, displaying all types of splicing alterations: exon skipping (*N* = 1404, 72.97%), splice site shift (*N* = 411, 21.36%), pseudo‐exon creation (*N* = 91, 4.73%) and intronic retention (*N* = 18, 0.94%) (Figure 2a and Supporting Information: Table S1). These spliceogenic variants occurred all along the gene sequence (Figure 2b). Consequently, our collection encompassed the diversity of splicing alterations and their underlying mechanisms.

Exon skipping was the most common splicing alteration (*N* = 1404, 72.97%), and causal variants were mainly located in the consensus splice site motifs (58.19%; 817/1404). Variants potentially impacting ESR motifs constituted the majority of alterations outside of consensus splice site motifs (38.68%; 543/1404). The remaining variants altered the BP or the PPT motifs, that is, 1.71% (24/1404) and 1.42% (20/1404), respectively.

Among the 411 variants (21.36%) causing a splice site shift, 59.12% (243/411) induced a partial exon deletion and 40.86% (168/411) induced a partial intron retention. These 411 variants included 204 variants (49.64%, 204/411) causing a shift in natural splice site position, and 207 (50.36%, 207/411) variants inducing the use of de novo/cryptic splice sites (see Section 2 for further explanation and Supporting Information: Figure S3). The shift of natural splice sites could occur regardless of the distance to the natural splice site (distance D1, see Supporting Information: Figure S3), with an average distance of 45 nucleotides. The de novo/cryptic splice sites were induced by variants within a range of 1–653 nucleotides from the natural splice site, with an average D1 distance of 48 nucleotides. The distance between these variants and their de novo/cryptic splice sites (distance D2, Supporting Information: Figure S3) was below 35 nucleotides for 99% of variants. The D2 median distance was one nucleotide, that is, the variants mapped within the canonical motif of the newly used splice site. Among the 207 variants which induced the use of de novo/cryptic splice sites, 102 (49.28%, 102/207) of them occurred in intronic sequences. An additional 91 intronic variants induced pseudo‐exon creation. The average size of these pseudo‐exons was 115 nucleotides with a range of 30–963 nucleotides. We found that the distance between the intronic variant and the natural splice site likely drives the two different impacts, as 97.06% (99/102) of spliceogenic variants located up to 150 nucleotides from the natural splice site led to a de novo/cryptic splice site, whereas 93.41% (85/91) of spliceogenic variants beyond 150 nucleotides induced the creation of a pseudo‐exon (Supporting Information: Figure S8). According to this result, the intronic regions beyond 150 nucleotides from the nearest exon were considered as deep intronic regions.

Lastly, 18 variants induced the retention of an entire intron. The average size of the retained intron was 775 nucleotides with a range of 75–3324 nucleotides. Half of these events (9/18; 50%) were observed for BP motif alterations.

The new collection of 426 variants had 53.8% (229/426) spliceogenic variants. The exon skipping was observed for 71.2% (163/229) of spliceogenic variants. The use of a new splice site was detected for 26.2% (60/229) of splicing alterations. The last six spliceogenic variants induced complex events such as the creation of new exons or the total intronic retention.

### 3.2 Distribution of variants within the comprehensive data collection

Using a Bayesian approach, we estimated a 2.08% (CI_95%_ [1.71%–2.53%]) probability of splicing alteration within our 227 genes, regardless of the variant position in the gene (see the Supporting Information: Methods section for details). In other words, a randomly selected variant has a probability of 2.08% to be spliceogenic in these genes. To be in line with this probability of splicing alteration within our 227 genes, we added 95,000 control variants in genes to our collection of 4616 variants, making a new data set of 99,616 variants (Figure 2c and Supporting Information: Table S1). Thus, among the 227 genes, the average number of variants was 439 (min: 1; max: 9083 variants) (Supporting Information: Table S2). The highest proportion of spliceogenic variants was obtained at exon/intron junctions up to 64.66% within the donor consensus sequence, whereas the lowest proportion was found for deep intronic regions, 0.102% (88/86,199) (Figure 2d). A proportion of 10.44% (639/6120) was found within the exons excluding variants in exonic splice site consensus motifs, clearly supporting the importance of ESR motifs in exon definition.

### 3.3 Metascore for detection of splice site creation or reinforcement (5′ss/3′ss de novo/cryptic)

We proposed a metascore model derived from the analysis of 213,413,039 splice sites, that is, 570,848 true positives from Ensembl (Zerbino et al., 2018) and 212,842,191 random AG/GT distinct of true positives splice sites. Training on 142,275,359 splice sites (two‐thirds of the data) showed that the combination of three scores (MES, SSF‐like, and ESR scores) significantly improved the model, *p* value (Wald test) < 10^−7^ (Supporting Information: Table S4). The validation phase using 71,137,680 splice sites (the remaining third of the data) confirmed that the use of the new metascore model outperformed the individual scores MES, SSF‐like, and ESR scores, with an area under the ROC curve of 0.971 versus 0.960, 0.933 and 0.768, respectively (Supporting Information: Figure S9).

### 3.4 SPiP model

Sixteen predictors were assessed to explain the spliceogenicity of a variant (Supporting Information: Table S5). Then, the backward variable selection gave us a final model with eight variables. The optimal number of predictors sampled for splitting at each node was 3 (Supporting Information: Table S6). The final random forest model of SPiP reached an average AUC of 0.986 on the validation set.

**Figure 3:**
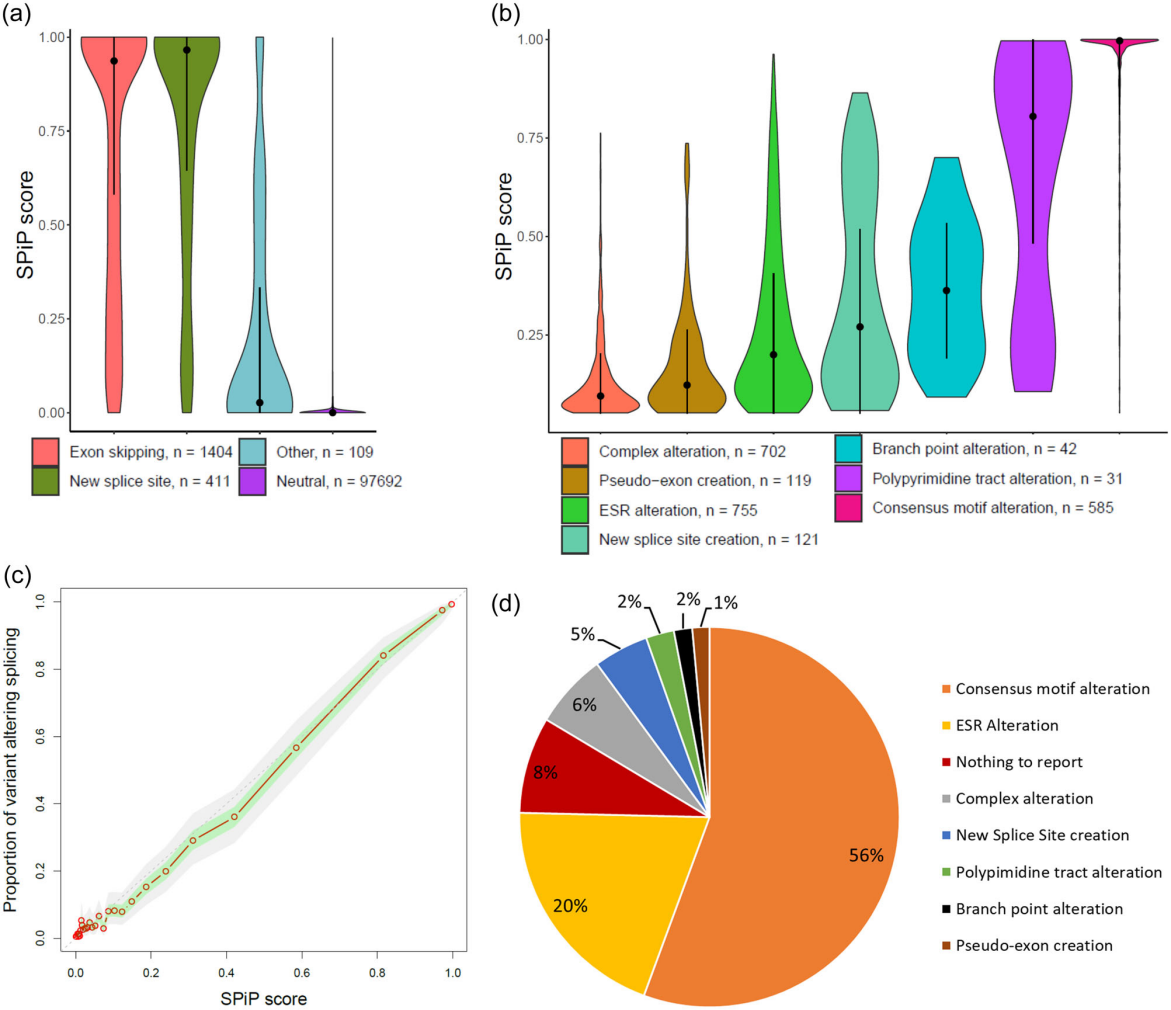
Evaluation of SPiP on the validation set. (a) Distribution of SPiP scores according to the impact of variants on splicing: exon skipping, new splice site use, other (pseudo‐exon and pseudo‐intron retention) and neutral, without impact on splicing (*N* = 99,616). Black points represent the median value and black lines represent the interquartile range. (b) Among variants with positive prediction, the distribution of SPiP score according to the altered motif: alteration of consensus motif, alteration of polypyrimidine tract, alteration of branch point motif, creation of new splice site, alteration of ESR motifs, creation of pseudo‐exon, and complex alterations (several motifs impacted simultaneously). Black points represent the median value and black lines represent the interquartile range. (c) Correlation between the SPiP score and the proportion of spliceogenic variants. (d) Proportion of variants that impact splicing according to their predictions. ESR, exonic splicing regulator; NTR, nothing to report; SPiP, Splicing Prediction Pipeline.

Among the validation set of variants, the highest values of SPiP score were observed for a variant inducing either exon skipping or the use of a new splice site, with a median SPiP score of 0.936 and 0.966, respectively. Consequently, these events were well predicted by the random forest model. The pseudo‐exon creation and the intronic retention presented a lower SPiP score (median of 0.026). Indeed, pseudo‐exon creation and intronic retention were rarely observed and so the model could not detect these events as well as exon skipping and/or use of new splice sites (Figure 3a).

We observed that the SPiP score trended to 1 when the consensus splice site motifs and the PPT were altered, with median SPiP scores of 0.996 and 0.804, respectively. The alteration of BP motifs, ESR motifs, and the use of a new splice site corresponded to intermediate values of SPiP score, median SPiP score of 0.358, 0.170, and 0.160, respectively. The lower values of SPiP score among the positive predictions were observed for the creation of a new splice site and for complex alterations (simultaneous alteration of multiple motifs), with median SPiP scores of 0.094 and 0.008, respectively (Figure 3b). This last category corresponded to several motifs potentially impacted by the variant and so represented a “gray area” in terms of prediction interpretation. The proportion of spliceogenic variants was correlated with the SPiP score. Indeed, the SPiP score showed a high correlation (*R* ^2^ = 0.9913) between the score value and the proportion of variants impacting splicing (Figure 3c). Thus, we could get a weighting on the probability that a variant impacts splicing according to the SPiP score.

The spliceogenic variants were mainly predicted as altering the consensus splice site motifs (56%). The alteration of ESR motifs explained another 20% of spliceogenic variants (Figure 3d). Variants with a prediction of complex alterations comprised 6% of the data. The less represented motif modifications were as follows: the use of new splice sites (5%), PPT (2%) and BP alterations (2%), and the creation of pseudo‐exons (1%). The false‐negative variants, spliceogenic variants predicted as NTR (nothing to report), represented 8% of spliceogenic variants.

### 3.5 Comparison of SPiP with SpliceAI and SQUIRLS

**Figure 4:**
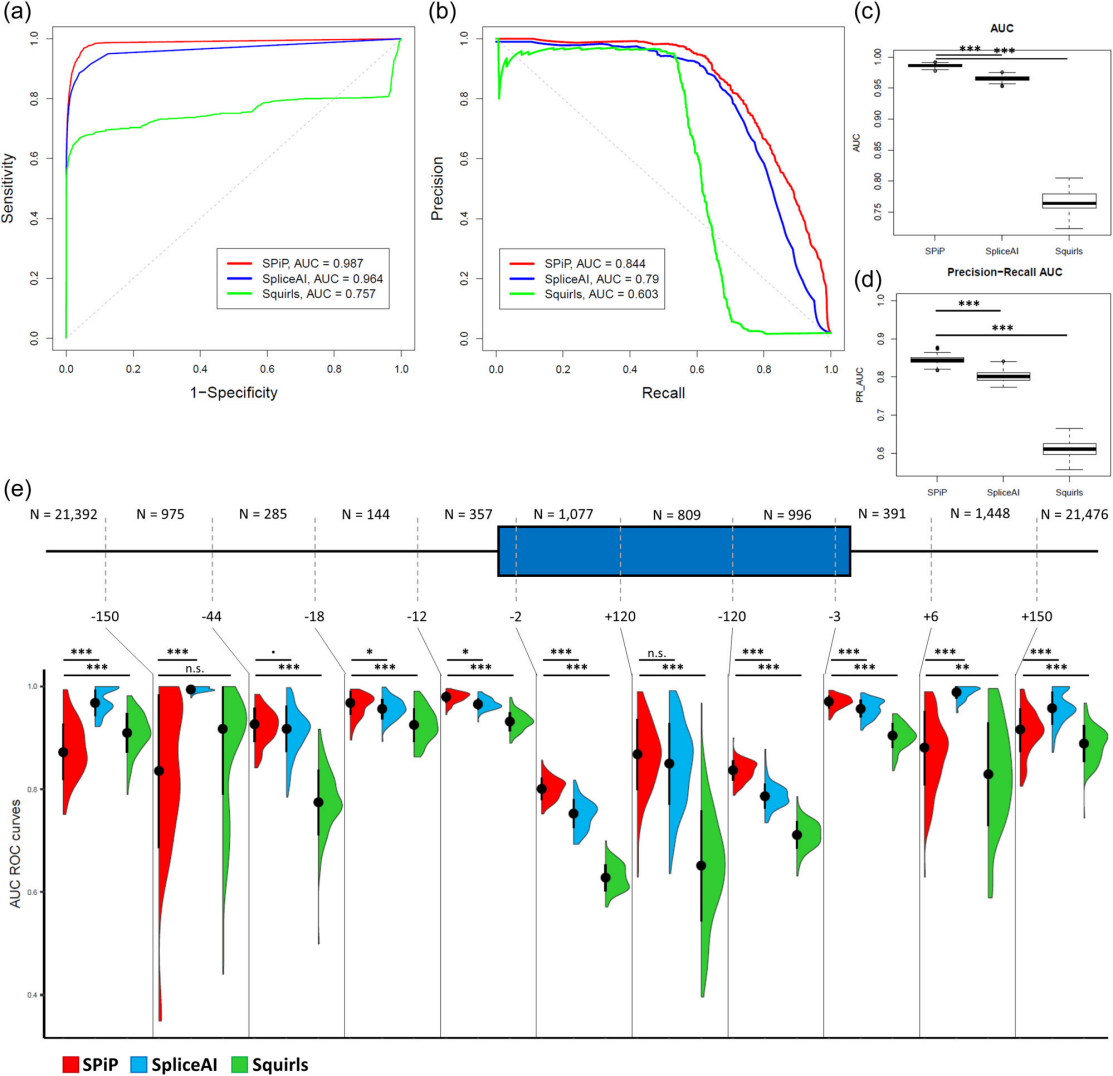
Comparison of SPiP with SpliceAI and SQUIRLS performances on the validation set (*N* = 49,350 variants, validation data set minus those not scored by SpliceAI). (a) ROC curves of SPiP, SpliceAI, and SQUIRLS for a particular iteration. (b) Precision‐recall curve of SPiP, SpliceAI, and SQUIRLS for a particular iteration. (c) Distribution of AUC values for the 100 iterations. (d) AUC of precision‐recall curve for 100 iterations. (e) Performance of SPiP (red), SpliceAI (blue), and SQUIRLS (green) measured by AUC of ROC curves all along the pre‐mRNA molecule for the 100 iterations. Black dots represent the median and black segments represent the interquartile range. AUC, area under the curve; n.s., not significant; ROC, receiver‐operating characteristic; SPiP, Splicing Prediction Pipeline. *p* < 0.1; *p < 0.05; **p < 0.01; ***p < 0.001.

On the complete data set of 99,616 variants, SpliceAI could not score 914 variants among them (0.9%) and only two of the 99,616 variants were not scored by SQUIRLS. For the data scored only by SPiP (*N* = 914 variants), we observed sensitivity and specificity of 91.84% and 93.29% (Supporting Information: Table S7). To ensure a fair comparison, we restricted the data set to the 98,702 variants scored by the three tools. Then, the comparison was done on 49,350 remaining variants (validation data set minus those not scored) randomly selected for each iteration. SPiP showed a better performance than SpliceAI and SQUIRLS for both ROC and precision‐recall analyses (Figure 4a,b). The 100 times iteration identified a significant improvement of variant prediction by SPiP compared to SpliceAI and SQUIRLS according to AUC of both ROC (0.986 vs, 0.965 and 0.766, respectively) and precision‐recall (0.847 vs. 0.799 and 0.612, respectively) curves (Figure 4c,d and Table 2). The comparison without the “control variants” showed also improved efficiency of SPiP compared to SpliceAI, with AUC 0.917 versus 0.900 (Supporting Information: Table S8). Thus, the set of “control variants” did not impact the comparison between SPiP and SpliceAI. Further, independently of a particular value of specificity or sensitivity, SPiP reached better values of sensitivity and precision (Supporting Information: Figure S10). SPiP outperformed SpliceAI for variants located in the BP area, the PPt, the consensus splice site, and in the entire exonic region. However, regarding the intronic regions, and in particular deep intronic regions, SpliceAI showed better performance than SPiP, beyond +6 nucleotides within 5′ part and −44 within 3′ part of intron (Figure 4e). Except for deep intronic variants close to the acceptor splice site, SPiP outperformed SQUIRLS, irrespective of the variant position.

**Table 2:** Performance of SPiP versus SpliceAI and SQUIRLS on the 99,616 variants from the complete set

	SPiP average [min; max]	SpliceAI average [min; max]	SQUIRLS average [min; max]
AUC	0.986*** [0.981; 0.991]	0.965 [0.959; 0.972]	0.766 [0.751; 0.788]
PR_AUC	0.847*** [0.828; 0.865]	0.799 [0.780; 0.822]	0.612 [0.578; 0.639]
Sensitivities for specificity at			
95%	95.87%*** [94.46%; 96.89%]	89.91% [88.63%; 91.47%]	68.12% [65.86%; 70.67%]
96%	94.55%*** [92.86%; 95.72%]	88.70% [87.31%; 90.51%]	67.31% [65.03%; 70.03%]
97%	92.52%*** [91.04%; 94.36%]	87.02% [84.20%; 89.13%]	66.08% [63.56%; 68.33%]
98%	89.37%*** [87.22%; 91.25%]	84.31% [82.80%; 86.67%]	64.72% [62.3%; 67.13%]
99%	83.73%*** [81.94%; 86.23%]	80.27% [78.06%; 83.26%]	61.71% [59.16%; 64.76%]
Precisions for recall at			
80%	69.82%*** [65.29%; 75.27%]	61.48% [55.25%; 68.49%]	2.31% [1.56%; 2.68%]
85%	58.45%*** [52.78%; 64.45%]	43.14% [37.71%; 53.01%]	1.67% [1.55%; 1.75%]
90%	44.66%*** [39.68%; 50.30%]	25.03% [20.31%; 32.15%]	1.76% [1.64%; 1.85%]
95%	29.93%*** [24.33%; 34.39%]	12.29% [6.72%; 15.62%]	1.83% [1.71%; 1.93%]

*Note*: Student test.

Abbreviations: AUC, area under the curve; SPiP, Splicing Prediction Pipeline.

* *p* < 0.05

** *p* < 0.01

*** *p* < 0.001.

**Figure 5:**
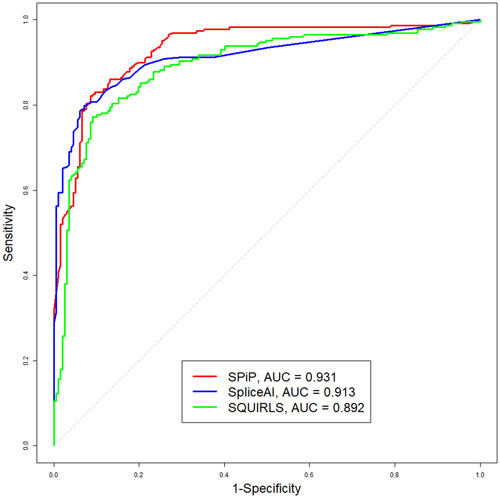
Performance of SPiP, SpliceAI, and SQUIRLS on the new collection of 426 variants

On the new collection of 426 variants, SPiP reached the best AUC value compared to SpliceAI and SQUIRLS, 0.931 vs. 0.913 and 0.892, respectively (Figure 5). SPiP revealed also the best values of sensitivity and accuracy compared to SpliceAI and SQUIRLS (86.0% vs. 84.7% and 82.1%). SPiP and SpliceAI had the same value of specificity: 85.3% (Table 3).

**Table 3:** Evaluation of SPiP, SpliceAI, and SQUIRLS on the new collection of 426 variants.

	SPiP, Th = 0.452	SpliceAI, Th = 0.12	SQUIRLS, Th = 0.018
Sensitivity	86.0% (197/229)	84.7% (194/229)	82.1% (188/229)
Specificity	85.3% (168/197)	85.3% (168/197)	82.7% (163/197)
Accuracy	85.7% (365/426)	85.0% (362/426)	82.4% (351/426)

Abbreviations: SPiP, Splicing Prediction Pipeline; Th, an optimal threshold used to compute sensitivity, specificity, and accuracy.

Lastly, parallel processing with SPiP gave an ~0.304 s/variant/CPU runtime under Linux Debian. SpliceAI showed a much slower runtime with ~7.63 s/variant/CPU. SQUIRLS was faster, with 0.014 s/variant/CPU runtime.

## 4 DISCUSSION

Owing to an international collaborative effort, we were able to collect 4616 variants scattered all along the sequence of 227 genes involved in 161 clinical signs and syndromes. This is one of the largest sets of curated, diagnostic‐grade data available to date, with their corresponding experimental RNA splicing data. This unique collection of curated splice data allowed a number of new observations on splicing anomalies. With the added “control variants,” we got a large collection of 99,616 variants mimicking a “real‐life” variant collection. It enabled us to develop SPiP within a random forest model, a splicing prediction suite powered by a combination of high‐performing selected bioinformatic tools plus a new score to detect de novo cryptic splice sites trained on 202,458,725 splice sites from Ensembl. As a result, SPiP performs in one single bioinformatics analysis, a comprehensive assessment of variant effects on different splicing motifs.

Using a comprehensive collection of 99,616 variants, we provided the first evaluation of prediction tools with a large proportion of nonspliceogenic variants (almost 98% of variants), mimicking a real‐life situation for analysis of whole‐genome sequencing data. However, this method was limited to the 227 genes included in our variant collection, and the results should be reevaluated to extend the analysis outside these genes. Under this condition, SPiP reached an AUC of 0.983 and 0.836 for ROC and precision‐recall analyses. A decision threshold for specificity at 99% gave an average sensitivity of 83.73% to detect exon skipping, use of a new splice site, pseudo‐exon creation, or intronic retention. The decision threshold for sensitivity at 85% provided an average precision of 58.45% (Table 2). These results highlighted the performance of SPiP to detect the versatility of splicing defects and maintained an acceptable level of false discovery rate (<50%). As a result, SPiP could be useful to detect spliceogenic variants among the huge amount of variants detected by next‐generation sequencing.

Among the diversity of prediction tools, we chose to compare SPiP to SpliceAI and SQUIRLS. In our data collection, SpliceAI had better performance to detect the creation of pseudo‐exons and usage of intronic de novo/cryptic splice site. However, SPiP outperformed SpliceAI to detect spliceogenic variants in exonic regions and for alteration of consensus splice site, PPT, and BP motifs. These differences in performance were also confirmed in the new collection of 426 variants. Despite the fact that none of these variants occurred at the same genomic position as the 99,616 variants used to develop SPiP and the majority of genes in the new collection did not have variants in the 99,616 variants. However, the number of variants was low compared to the collection of 99,616 variants. As a result, we cannot show significant differences in performances on this new collection of 426 variants. SpliceAI was trained on Gencode transcript data (Frankish et al., 2019) and so the tool was trained to detect splice site usage (Jaganathan et al., 2019). By contrast, SPiP was trained on a clinical grade variant collection where the majority of spliceogenic variants induced exon skipping (73% of spliceogenic variants). These differences in the training process could explain the observed differences between the two tools.

We also observed that both prediction tools had a better overall performance for nondeep intronic (i.e., located between 150 first and last nucleotide of the intron) variants than for exonic variants. Within exonic regions, spliceogenic variants could create new splice sites but mostly induced exon skipping by disrupting ESR motifs. The diversity and low conservation of ESR motifs (Rosenberg et al., 2015) make them hard to predict whatever the prediction tool. A major limitation of these tools seems to be the prediction of spliceogenic changes among deep intronic variants (*N* = 86,199 variants). Spliceogenic variants were particularly rare; 0.102% (88/86,199) in our collection. This paradigm showed that even if SpliceAI had the optimal performance for these regions with a high specificity of 99.77% (85,920/86,111) for a sensitivity of 79.55% (70/88), the false discovery rate was 75.48% (191/261). Thus identifying spliceogenic variants in these regions is like “looking for a needle in a haystack.” However, we believe that the growing implementation of high‐output functional assays (Adamson et al., 2018; Casadei et al., 2019; Cheung et al., 2019; Davy et al., 2017) could in the future solve these issues. In particular, the extension of these functional assays to deep intronic variants would be a new opportunity for the molecular diagnosis.

SQUIRLS showed the lowest overall performance. This could be explained by the training set of SQUIRLS, limited to ClinVar variants with clinically confirmed impact. Using these data could introduce a bias as a significant proportion of variants of unknown significance impact splicing and they were not taken into account by SQUIRLS (Truty et al., 2021).

To promote the accessibility of SPiP, we have developed two versions of this tool, one for Windows (https://sourceforge.net/projects/splicing-prediction-pipeline/) and the other for Linux OS (https://github.com/raphaelleman/SPiP). The Windows version offers a user‐friendly interface (Supporting Information: Figure S11) and the Linux version allows parallel processing of a great number of variants to meet the needs of whole‐exome or even whole‐genome sequencing. By providing information about probably altered splicing motifs and CIs for a given SPiP score (Supporting Information: Figure S11), the output of SPiP can help variant interpretation and prioritization of in vitro RNA studies. This information helps to reduce the “black box” effect, which is specific to the machine learning approaches.

SPiP predictions could also be helpful for variant classification (Richards et al., 2015), but rules should be defined by expert consortia.

Overall, SPiP provides a complete prediction solution and lends itself to a unique suite for massive prediction, to be used in the context of variant interpretation in genomic medicine.

## CONFLICT OF INTEREST

H. T. was employed by Interactive Biosoftware for the time period October 2015–September 2018 in the context of a public–private PhD project (CIFRE fellowship #2015/0335) partnership between INSERM and Interactive Biosoftware. The remaining authors declare no conflict of interest.

## Supporting information

Supporting information.

Supporting information.

Supporting information.
