# Supplementary material for: SPiP: Splicing Prediction Pipeline, a machine learning tool for massive detection of exonic and intronic variant effects on mRNA splicing
Source: Hum Mutat. 2022 Nov 20;43(12):2308–23. doi: 10.1002/humu.24491 (PMC10946553; doi:10.1002/humu.24491)
Supplement: Supplementary file 1 — Supporting information. [file HUMU-43-2308-s001.docx]

# **SUPPLEMENTARY MATERIALS - SPiP: Splicing Prediction Pipeline, a machine learning tool for massive detection of exonic and intronic variant effect on mRNA splicing.**

INDEX

[**SUPPLEMENTARY MATERIALS** 1](#_Toc103247017)

[**SUPPLEMENTARY METHODS** 2](#_Toc103247018)

[**Selection of optimal tools according to the benchmark studies** 2](#_Toc103247019)

[**Estimation of splicing probability** 3](#_Toc103247020)

[**SUPPLEMENTARY TABLES** 9](#_Toc103247021)

[**Supplementary Table S2:** Distribution of the complete collection of variant among the 227 genes 10](#_Toc103247022)

[**Supplementary Table S3:** Parameters of metascore model 17](#_Toc103247023)

[**Supplementary Table S4:** Selection of predictors for Random Forest model. 18](#_Toc103247024)

[**Supplementary Table S5:** Selection of optimal number of predictors 19](#_Toc103247025)

[**Supplementary Table S6:** SPiP efficiency on SpliceAI missing data 20](#_Toc103247026)

[**Supplementary Table S7:** Performance of SPiP versus SpliceAI and SQUIRLS. 21](#_Toc103247027)

[**SUPPLEMENTARY FIGURES** 22](#_Toc103247028)

[**Supplementary** **Figure S1:** Illustration of variant-induced splicing alterations. 22](#_Toc103247029)

[**Supplementary** **Figure S2:** Splicing mechanism and motifs. 23](#_Toc103247030)

[**Supplementary** **Figure S3:** Mechanisms inducing the use of new splice site. 24](#_Toc103247031)

[**Supplementary** **Figure S4:** Collection of 4,616 variants with RNA *in vitro* studies. 25](#_Toc103247032)

[**Supplementary Figure S5:** Workflow to get a set of data representative of the natural distribution of spliceogenic variants. 26](#_Toc103247033)

[**Supplementary Figure S6:** The strategy used by SPiP to detect cryptic splice 27](#_Toc103247034)

[**Supplementary Figure S7:** Pipeline of SPiP to detect which motif is probably impacted by a variant. 28](#_Toc103247035)

[**Supplementary** **Figure S8:** Distance between intronic variants and their nearest natural splice sites discriminates between variant-induced splice site shifts and pseudo-exons usage. 29](#_Toc103247036)

[**Supplementary Figure S9:** Prediction of splice site creation or reinforcement. 30](#_Toc103247037)

[**Supplementary Figure S10:** Comparison between SPiP, SpliceAI and SQUIRLS for the 100 iterations, n = 49,350 variants. 31](#_Toc103247038)

[**Supplementary Figure S11:** Screenshot of SPiP console for Windows version and an example of SPiP outputs. 32](#_Toc103247039)

[**REFERENCES** 33](#_Toc103247040)

## **SUPPLEMENTARY METHODS**

### **Selection of optimal tools according to the benchmark studies**

#### Consensus splice site

As distinct 5’/3’ ss prediction tools are based on distinct consensus motif lengths, we defined the smallest overlap consensus length to ensure homogeneous comparison: -3; +6 for donor motif length and -12; +2 for acceptor motif length. We took our previous benchmark study of Leman *et al.* (Leman et al., 2018), as this work was one of latest and largest *in silico* studies of splice variants with their corresponding *in vitro/ex vivo* transcript analyses conducted by a consortium (n = 253 variants in 11 genes). We compared Splice Site Finder (SSF) (Shapiro and Senapathy, 1987), Neural Network Splice (NNS) (Reese et al., 1995), GeneSplicer (GS) (Pertea et al., 2001), MaxEntScan (MES) (Yeo and Burge, 2004), Human Splicing Finder (HSF) v3.0 (Desmet et al., 2009), and Splicing Prediction in Consensus Element (SPiCE) (Leman et al., 2018). These tools give quantitative values and we calculated the score variation between wild-type and variant sequences.

#### Polypyrimidine tract

The PPT was defined between the 13^th^ and the 17^th^ nucleotide (included) upstream of the 3’ splice site (-17 to -13 intronic region). The reason is that the minimal common size of 3’ splice site predictors extends up to 12 nucleotides in the intron. The branch point area was described as starting from 18^th^ nucleotide in the intron (Mercer et al., 2015). To address analysis of a variants occurring in the PPT region, we used the benchmark studies of Leman *et al.* (Leman et al., 2018) and Houdayer *et al.,* (Houdayer et al., 2012) to compare NNS, GS and MES.

MES score was used according to the guidelines from the Unicancer Genetic Group in 2012 (Houdayer et al., 2012) on variants occurring in consensus splice sites. Indeed, a decision-making threshold, corresponding to delta score at -15 % $\left( \frac{\left( MES_{mutated}-MES_{wildtype} \right)}{MES_{wildtype}} \right)$ was proposed to predict splicing alteration.

#### Branch point:

The optimal tool for branch point prediction was defined on a set of variants with their RNA *in vitro* studies occurring in the branch point area (-18 to -44) (Mercer et al., 2015), by our recently published work (Leman et al., 2020). This previous study benchmarked 6 BP-dedicated tools: HSF (Desmet et al., 2009), SVM-BPfinder (Corvelo et al., 2010), Branch Point Prediction (BPP) (Zhang et al., 2017), Branchpointer (Signal et al., 2018), LaBranchoR (Paggi and Bejerano, 2018) and RNA Branch Point Selection (RNABPS) (Nazari et al., 2019). Briefly, we assayed two strategies to predict variant alteration of branch points: i) is a variant located in a predicted branch point motif and ii) is the variant decreasing the score of a predicted branch point.

#### Exonic Splicing Regulators (ESR)

The optimal tools for ESR analysis were selected based on findings from previous publications. The first one (Soukarieh et al., 2016) compared QUEPASA (Giacomo et al., 2013), ∆HZ_EI_ (Erkelenz et al., 2014) and SPANR (Xiong et al., 2015) especially on 22 variants occurring in exon 10 of the *MLH1* gene. Another two benchmark studies performed by Grodecka *et al.,* (Grodecká et al., 2017b, 2017a) compare to QUEPASA, EX-SKIP (Raponi et al., 2011), ∆HZ_EI_ and SPANR.

### **Estimation of splicing probability**

We needed to generate a variant collection mimicking real life, *i.e.* without collection bias. To reach this goal, we calculated the *a priori* probability that a variant impacts splicing ($P(S)$) from a Bayesian approach and defined by the formula:

$$\begin{aligned} \mathbf{P}\left( \mathbf{S} \right)\mathbf{=}\frac{\mathbf{S}}{\mathbf{N}}\boldsymbol{\#}\left( \boldsymbol{SEQ Equation \backslash* ARABIC}\mathbf{1} \right) \end{aligned}$$

Where $S$ was the number of spliceogenic variants, $N$ the total number of variants. Thus if $S$ corresponded to the spliceogenic variants from our collection, the number $N$ should be corrected to be in line with $P\left( S \right)$. We can define the number $N^{corr}$ of negative variants, *i.e.* without impact on splicing, to add to our variant collection ($N^{obs}$).

$$\begin{aligned} \mathbf{N=}\mathbf{N}^{\mathbf{obs}}\mathbf{+}\mathbf{N}^{\mathbf{corr}}\boldsymbol{\#}\left( \boldsymbol{SEQ Equation \backslash* ARABIC}\mathbf{2} \right) \end{aligned}$$

Thus we can calculate $N^{\mathrm{corr}}$ from (1) and (2):

$$\begin{aligned} \mathbf{N}^{\mathbf{corr}}\mathbf{=}\frac{\mathbf{S}}{\mathbf{P}\left( \mathbf{S} \right)}\mathbf{-}\mathbf{N}^{\mathbf{obs}}\#\left( SEQ Equation \backslash* ARABIC 3 \right) \end{aligned}$$

To solve equation (3), we used this Bayesian approach to associate $P\left( S \right)$ with the exonic data (4). As explained previously, the exonic regions were the most studied.

$$\begin{aligned} \mathbf{P}\left( \mathbf{S} | \mathbf{E} \right)\mathbf{=}\frac{\mathbf{P}\left( \mathbf{E} | \mathbf{S} \right)\boldsymbol{\times P}\left( \mathbf{S} \right)}{\mathbf{P}\left( \mathbf{E} \right)}\boldsymbol{\#}\left( \boldsymbol{SEQ Equation \backslash* ARABIC}\mathbf{4} \right) \end{aligned}$$

Where $E$ is the event variant in an exonic region and $S$ is the event of splicing alteration. Thus, the $P\left( E | S \right)$ term is the probability that a spliceogenic variant occurs within an exon, $P\left( S | E \right)$ the proportion of spliceogenic variants among exonic variants, and $P\left( E \right)$ the natural proportion of exonic variants among intra-genic variants. From equation (4), we can define $P\left( S \right)$ by:

$$\begin{aligned} \mathbf{P}\left( \mathbf{S} \right)\mathbf{=P}\left( \mathbf{E} \right)\boldsymbol{\times}\frac{\mathbf{P}\left( \mathbf{S} | \mathbf{E} \right)}{\mathbf{P}\left( \mathbf{E} | \mathbf{S} \right)}\boldsymbol{\#}\left( \boldsymbol{SEQ Equation \backslash* ARABIC}\mathbf{5} \right) \end{aligned}$$

#### Prerequisites to calculate $P\left( S \right)$

According to the equation (5) to estimate the value of $P\left( S \right)$, we need the reliable value for the terms $P\left( E | S \right)$, $P\left( S | E \right)$, and $P\left( E \right)$.

$P\left( E \right)$ represented the natural proportion of an exonic variants among the intra-genic variants. Consequently, this estimation implied data from an unbiased population. The 1000 Genomes project (The 1000 Genomes Project Consortium, 2015) sequenced the whole genome of 2,500 people from the healthy world population. So the data from the 1000 Genomes project suited the criteria to estimate $P\left( E \right)$.

$P\left( S | E \right)$ corresponded to the difference of splicing alteration mechanisms by a variant between the exonic and non exonic (*i*.e. intron) regions, inside the pre-mRNA molecule. Then, the data, to estimate $P\left( S | E \right)$, should be representative of the mechanism diversity of splicing alterations by a variant. As we gathered a wide variety of spliceogenic variants, we used our data to estimate this term.

$P\left( E | S \right)$ was the risk that an exonic variant impacts splicing. Several studies had proposed an estimation of this risk (Teraoka et al., 1999; Tournier et al., 2008; Lim et al., 2011; Sterne-Weiler et al., 2011; Mueller et al., 2015; Soemedi et al., 2017). As a result, we were confronted with a wide range of risk according to the study, with an average of 23.5 % and a range of 10.3 % – 48.0 %:

- Study of ATM variant by RT-PCR of LCLs (Exon+Consensus); Number of spliceogenic variants: 30/62 (48.0%), (Teraoka et al., 1999)
- Minigene splicing assays (MLH1 and MSH2); Number of spliceogenic variants: 13/67 (19.4%), (Tournier et al., 2008)
- Simulated mutation from HGMD and intraallelic L1 Distance; Number of spliceogenic variants: -/- (22.0%), (Lim et al., 2011)
- Prediction of loss ESE/gain ESS of 27,681 HGMD mutations with 83 spliced minigene assays for validation; Number of spliceogenic variants: 7,154/27,681 (25.8%), (Sterne-Weiler et al., 2011)
- Minigene splicing assays (SMN1); Number of spliceogenic variants: 32/138 (23.2%), (Mueller et al., 2015)
- Massively parallel splicing assay (MaPSy); Number of spliceogenic variants: 513/4964 (10.3%), (Soemedi et al., 2017)

To assess the adequacy of our observed proportion of spliceogenic variants with the sum of literature data, we calculated the probability that the observed proportion was in line with the literature data. We considered that event of splicing alteration as a Bernoulli experiment. Thus, the probability of splicing alteration in exon could be adjusted to binomial distribution.

$$\begin{aligned} \boldsymbol{K\sim}\mathbf{Bin}_{\mathbf{i}}\left( \mathbf{n}_{\mathbf{i}}\mathbf{,}\mathbf{p}_{\mathbf{i}} \right)\boldsymbol{\#}\left( \boldsymbol{SEQ Equation \backslash* ARABIC}\mathbf{6} \right) \end{aligned}$$

Where $K$ is the number of spliceogenic variants, $n_{i}$ is the number of variants in the $i^{th}$ study and $p_{i}$ is the proportion of spliceogenic variants. As each study reported different proportions of spliceogenic variants in exons from differents collections of variants, we proposed a weighting factor $\omega$ based on the number of variants.

$$\begin{aligned} \boldsymbol{\omega}_{\mathbf{i}}\mathbf{=}\frac{\mathbf{n}_{\mathbf{i}}}{\sum_{\mathbf{i}} \mathbf{n}_{\mathbf{i}}}\boldsymbol{\#}\left( \boldsymbol{SEQ Equation \backslash* ARABIC}\mathbf{7} \right) \end{aligned}$$

Thus, the probability to observe $k$ spliceogenic variants according to the literature data is:

$$\begin{aligned} \mathbf{P}\left( \boldsymbol{x\geq k} \right)\mathbf{=1-}\sum_{\mathbf{i=1}} \boldsymbol{\omega}_{\mathbf{i}}\left( \sum_{\mathbf{j=k}}^{\mathbf{n}} \mathbf{P}_{\mathbf{i}}\left( \mathbf{x=j} \right) \right)\boldsymbol{\#}\left( \boldsymbol{SEQ Equation \backslash* ARABIC}\mathbf{8} \right) \end{aligned}$$

And according to (6), the term $P_{i}\left( x=j \right)$, is:

$$\begin{aligned} \mathbf{P}_{\mathbf{i}}\left( \mathbf{x=j} \right)\mathbf{=}\left( \begin{matrix} \mathbf{n} \\ \mathbf{j} \end{matrix} \right){\mathbf{p}_{\mathbf{i}}}^{\mathbf{j}}\left( \mathbf{1-}\mathbf{p}_{\mathbf{i}} \right)^{\mathbf{n-j}}\boldsymbol{\#}\left( \boldsymbol{SEQ Equation \backslash* ARABIC}\mathbf{9} \right) \end{aligned}$$

The probability that the number of spliceogenic variants in exons suits the literature data, according to (8) and (9), is:

$$\begin{aligned} \mathbf{P}\left( \boldsymbol{x\geq k} \right)\mathbf{= 1-}\sum_{\mathbf{i=1}} \boldsymbol{\omega}_{\mathbf{i}}\left( \sum_{\mathbf{j=k}}^{\mathbf{n}} \left( \begin{matrix} \mathbf{n} \\ \mathbf{j} \end{matrix} \right){\mathbf{p}_{\mathbf{i}}}^{\mathbf{j}}\left( \mathbf{1-}\mathbf{p}_{\mathbf{i}} \right)^{\mathbf{n-j}} \right)\boldsymbol{\#}\left( \boldsymbol{SEQ Equation \backslash* ARABIC}\mathbf{10} \right) \end{aligned}$$

Once that this calculated probability, equation (10), was above 5 %, we were able to calculate $P\left( S \right)$.

#### Calculation of $P\left( S \right)$

$P\left( E | S \right)$ and $P\left( S | E \right)$ were estimated from our variant collection, *i.e.* we used the observed variant distribution in this collection, with $S^{exon}$, the number of exonic variant impacting splicing, $N^{{obs}_{exon}}$, the total number of exonic variants, $N^{{obs}_{spliceogenic}}$, the total number of variant impacting splicing. The canonical variants (+1; +2 / -1; -2) were excluded due to their heavy impact on splicing.

$$\begin{aligned} \mathbf{P}\left( \mathbf{S} | \mathbf{E} \right)\mathbf{=}\frac{\mathbf{S}^{\mathbf{exon}}}{\mathbf{N}^{\mathbf{obs}_{\mathbf{exon}}}}\boldsymbol{\#}\left( \boldsymbol{SEQ Equation \backslash* ARABIC}\mathbf{11} \right) \end{aligned}$$

$$\begin{aligned} \mathbf{P}\left( \mathbf{E} | \mathbf{S} \right)\mathbf{=}\frac{\mathbf{S}^{\mathbf{exon}}}{\mathbf{N}^{\mathbf{obs}_{\mathbf{spliceogenic}}}}\boldsymbol{\#}\left( \boldsymbol{SEQ Equation \backslash* ARABIC}\mathbf{12} \right) \end{aligned}$$

Thus, from (11) and (12), the term $\frac{P\left( S | E \right)}{P\left( E | S \right)}$ could be simplify in

$$\begin{aligned} \frac{\mathbf{P}\left( \mathbf{S} | \mathbf{E} \right)}{\mathbf{P}\left( \mathbf{E} | \mathbf{S} \right)}\mathbf{=}\frac{\mathbf{N}^{\mathbf{obs}_{\mathbf{spliceogenic}}}}{\mathbf{N}^{\mathbf{obs}_{\mathbf{exon}}}}\boldsymbol{\#}\left( \boldsymbol{SEQ Equation \backslash* ARABIC}\mathbf{13} \right) \end{aligned}$$

And thus, from the equations (5) and (13) we defined $P\left( S \right)$ as:

$$\begin{aligned} \mathbf{P}\left( \mathbf{S} \right)\mathbf{=P}\left( \mathbf{E} \right)\mathbf{*}\frac{\mathbf{N}^{\mathbf{obs}_{\mathbf{spliceogenic}}}}{\mathbf{N}^{\mathbf{obs}_{\mathbf{exon}}}}\boldsymbol{\#}\left( \boldsymbol{SEQ Equation \backslash* ARABIC}\mathbf{14} \right) \end{aligned}$$

According to (5) and (14), we can calculate $N^{\mathrm{corr}}$.

#### Definition of the number of negative variants to add

The variants used in $N^{\mathrm{corr}}$ were derived from frequent variants (Minor Allele Frequency, MAF > 5 %) occurring in genes described in our collection data. As the variants in our collection were studied for diagnostic purposes, the genes described in this set were involved in human diseases. Therefore, we hypothesized that frequent (MAF > 5 %) variants cannot alter the functionality of these genes and so have no major impact on splicing. Our set of data plus frequent variants permitted estimation of the probabilities of splicing alteration according to the SPiP prediction and the position in transcript, *i.e.* the positive predictive values and the negative predictive values.

For each estimated values of probabilities, we calculated the 95 % confidence interval according to the Wilson method (Agresti and Coull, 1998):

$$\begin{aligned} \mathbf{IC}_{\boldsymbol{1-\alpha}}\mathbf{=}\left[ \left( \frac{\mathbf{P+}\frac{\mathcal{z}_{\frac{\boldsymbol{\alpha}}{\mathbf{2}}}^{\mathbf{2}}}{\mathbf{2n}}}{\mathbf{1+}\frac{\mathcal{z}_{\frac{\boldsymbol{\alpha}}{\mathbf{2}}}^{\mathbf{2}}}{\mathbf{n}}} \right)\boldsymbol{\pm}\frac{\mathcal{z}_{\frac{\boldsymbol{\alpha}}{\mathbf{2}}}\sqrt{\frac{\mathbf{P}\left( \mathbf{1-P} \right)\mathbf{+}\frac{\mathcal{z}_{\frac{\boldsymbol{\alpha}}{\mathbf{2}}}^{\mathbf{2}}}{\mathbf{4n}}}{\mathbf{n}}}}{\mathbf{1+}\frac{\mathcal{z}_{\frac{\boldsymbol{\alpha}}{\mathbf{2}}}^{\mathbf{2}}}{\mathbf{n}}} \right]\boldsymbol{\#}\left( \boldsymbol{SEQ Equation \backslash* ARABIC}\mathbf{15} \right) \end{aligned}$$

where $\mathcal{z}_{\frac{\alpha}{2}}$, denotes the $1-\alpha$ quantile of the standard normal distribution.

#### Models application

These values were obtained on a selected set of data, 1,924 spliceogenic variants ($S$) among a total of 4,616 variants ($N^{obs}$) (Supplementary Table S1). However, to mimic realistic genomic situations and to calculate the probability of splicing alteration ($P\left( S \right)$), we reasoned that an additional set of common variants presumably without effect on splicing should be added ($N^{\mathrm{corr}}$). The estimation of $N^{\mathrm{corr}}$ and therefore $P\left( S \right)$ was obtained by a Bayesian approach (see “method” section for details). We observed 826 exonic spliceogenic variants ($S^{exon}$) among 3,090 exonic variants ($N^{{obs}_{exon}}$) in our collection of data, *i.e.* 26.73 % ($P\left( S | E \right)$). Excepting the 3’/5’ most extreme exonic portion, $P\left( S | E \right)$ appeared to be constant within the body of the exons (data not shown).

This proportion was not significantly different from the proportion reported in the literature (Table 1), ranging from 10.3 % – 48.0 % and p-value of 0.138 (see “Methods” section for details). A total of 1,313 spliceogenic variants, excepted canonical variants (+1; +2 / -1; -2, n = 613 variants), ($N^{{obs}_{spliceogenic}}$), were in our collection of variants. Among them, 62.60 % (822/1,313) were exonic ($P\left( E | S \right)$). From the 1000 Genome project (download April 4, 2019), we downloaded 38,529,509 intragenic variants, of whom 1,884,730 were exonic ($P\left( E \right)$ = 4.89 %). As a result, the Bayesian probability that a variant alters splicing whatever its position in a gene ($P\left( S \right)$) was 2.08 % (CI_95%_ [1.71 % – 2.53 %]). As we had 1,924 spliceogenic variants (S) among a total of 4,616 variants ($N^{obs}$), to be in line with this 2.08 % (CI_95%_ [1.71 % – 2.53 %]) value, we needed to add between 76,047 (2.53 %) and 112,515 (1.71 %) non-spliceogenic variants. Consequently, 103,568 variants, with MAF > 5 %, from our 227 genes were extracted from UCSC (31) (download March 11, 2019), of whom 95,000 ($N^{corr}$) were randomly selected (Supplementary Table S2) and added to the evaluation set, thus enabling a 1.93 % of spliceogenic variants in this “real life” evaluation set. On this new set of data (n = 99,616 variants) and in agreement with the literature (p-value = 0.15), we observed that 1.18 % (1,098/93,186) of intronic variants and 12.85 % (826/6,430) of exonic variants impacted splicing, respectively

## **SUPPLEMENTARY TABLES**

Table S1 and Table S3 are in excel format.

### **Supplementary Table S2:** Distribution of the complete collection of variant among the 227 genes with the clinical signs and syndromes if alteration (source: [www.orpha.net](http://www.orpha.net)). NA: no particular syndrome linked to gene alteration

| Gene  N = 227 | Variants  N = 99,616 | Clinical Signs and Syndromes  N = 161 | Cancerology  N = 26 | Development  N = 52 | metabolomics and muscular-skeletal disorders  N = 67 | Neurology, Immunology and Hematology disorders  N = 41 | No-syndromic genes  N = 41 |
| --- | --- | --- | --- | --- | --- | --- | --- |
| *ABCB11* | 703 | [Benign recurrent intrahepatic cholestasis type 2](https://www.orpha.net/consor/cgi-bin/Disease_Search.php?lng=EN&data_id=14534&MISSING%20CONTENT=Benign-recurrent-intrahepatic-cholestasis-type-2&search=Disease_Search_Simple&title=Benign%20recurrent%20intrahepatic%20cholestasis%20type%202) |  |  | X |  |  |
| *ABCC8* | 515 | [Autosomal dominant hyperinsulinism due to SUR1 deficiency](https://www.orpha.net/consor/cgi-bin/Disease_Search.php?lng=EN&data_id=20384&MISSING%20CONTENT=Autosomal-dominant-hyperinsulinism-due-to-SUR1-deficiency&search=Disease_Search_Simple&title=Autosomal%20dominant%20hyperinsulinism%20due%20to%20SUR1%20deficiency) |  |  | X |  |  |
| *ACACB* | 616 | NA |  |  |  |  | X |
| *ACADM* | 286 | [Medium chain acyl-CoA dehydrogenase deficiency](https://www.orpha.net/consor/cgi-bin/Disease_Search.php?lng=EN&data_id=3570&MISSING%20CONTENT=Medium-chain-acyl-CoA-dehydrogenase-deficiency&search=Disease_Search_Simple&title=Medium%20chain%20acyl-CoA%20dehydrogenase%20deficiency) |  |  | X |  |  |
| *ACADSB* | 250 | [2-methylbutyryl-CoA dehydrogenase deficiency](https://www.orpha.net/consor/cgi-bin/Disease_Search.php?lng=EN&data_id=11183&MISSING%20CONTENT=2-methylbutyryl-CoA-dehydrogenase-deficiency&search=Disease_Search_Simple&title=2-methylbutyryl-CoA%20dehydrogenase%20deficiency) |  |  | X |  |  |
| *ACAT1* | 92 | [Beta-ketothiolase deficiency](https://www.orpha.net/consor/cgi-bin/Disease_Search.php?lng=EN&data_id=713&MISSING%20CONTENT=Beta-ketothiolase-deficiency&search=Disease_Search_Simple&title=Beta-ketothiolase%20deficiency) |  |  | X |  |  |
| *ADA* | 152 | NA |  |  |  |  | X |
| *AGBL1* | 5389 | [Fuchs endothelial corneal dystrophy](https://www.orpha.net/consor/cgi-bin/Disease_Search.php?lng=EN&data_id=13991&MISSING%20CONTENT=Fuchs-endothelial-corneal-dystrophy&search=Disease_Search_Simple&title=Fuchs%20endothelial%20corneal%20dystrophy) |  | X |  |  |  |
| *ALDH3A2* | 114 | [Sjögren-Larsson syndrome](https://www.orpha.net/consor/cgi-bin/Disease_Search.php?lng=EN&data_id=586&MISSING%20CONTENT=Sjogren-Larsson-syndrome&search=Disease_Search_Simple&title=Sj%F6gren-Larsson%20syndrome) |  |  |  | X |  |
| *ALG3* | 18 | [ALG3-CDG](https://www.orpha.net/consor/cgi-bin/Disease_Search.php?lng=EN&data_id=11347&MISSING%20CONTENT=ALG3-CDG&search=Disease_Search_Simple&title=ALG3-CDG) |  |  |  | X |  |
| *ALPL* | 422 | [Adult hypophosphatasia](https://www.orpha.net/consor/cgi-bin/Disease_Search.php?lng=EN&data_id=19535&MISSING%20CONTENT=Adult-hypophosphatasia&search=Disease_Search_Simple&title=Adult%20hypophosphatasia) |  |  | X |  |  |
| *APC* | 418 | [APC-related attenuated familial adenomatous polyposis](https://www.orpha.net/consor/cgi-bin/Disease_Search.php?lng=EN&data_id=19550&MISSING%20CONTENT=APC-related-attenuated-familial-adenomatous-polyposis&search=Disease_Search_Simple&title=APC-related%20attenuated%20familial%20adenomatous%20polyposis) | X |  |  |  |  |
| *AR* | 296 | NA |  |  |  |  | X |
| *ATM* | 530 | [Ataxia-telangiectasia](https://www.orpha.net/consor/cgi-bin/Disease_Search.php?lng=EN&data_id=104&MISSING%20CONTENT=Ataxia-telangiectasia&search=Disease_Search_Simple&title=Ataxia-telangiectasia) |  |  |  | X |  |
| *ATP6AP2* | 93 | [X-linked intellectual disability, Hedera type](https://www.orpha.net/consor/cgi-bin/Disease_Search.php?lng=EN&data_id=12510&MISSING%20CONTENT=X-linked-intellectual-disability--Hedera-type&search=Disease_Search_Simple&title=X-linked%20intellectual%20disability,%20Hedera%20type) |  | X |  |  |  |
| *ATP7A* | 130 | [Occipital horn syndrome](https://www.orpha.net/consor/cgi-bin/Disease_Search.php?lng=EN&data_id=7035&MISSING%20CONTENT=Occipital-horn-syndrome&search=Disease_Search_Simple&title=Occipital%20horn%20syndrome) |  |  | X |  |  |
| *ATR* | 409 | [Familial cutaneous telangiectasia and oropharyngeal cancer predisposition syndrome](https://www.orpha.net/consor/cgi-bin/Disease_Search.php?lng=EN&data_id=21457&MISSING%20CONTENT=Familial-cutaneous-telangiectasia-and-oropharyngeal-cancer-predisposition-syndrome&search=Disease_Search_Simple&title=Familial%20cutaneous%20telangiectasia%20and%20oropharyngeal%20cancer%20predisposition%20syndrome) | X |  |  |  |  |
| *BCKDHA* | 183 | [Classic maple syrup urine disease](https://www.orpha.net/consor/cgi-bin/Disease_Search.php?lng=EN&data_id=20168&MISSING%20CONTENT=Classic-maple-syrup-urine-disease&search=Disease_Search_Simple&title=Classic%20maple%20syrup%20urine%20disease) |  |  | X |  |  |
| *BRCA1* | 969 | [Hereditary breast and ovarian cancer syndrome](https://www.orpha.net/consor/cgi-bin/Disease_Search.php?lng=EN&data_id=3384&MISSING%20CONTENT=Hereditary-breast-and-ovarian-cancer-syndrome&search=Disease_Search_Simple&title=Hereditary%20breast%20and%20ovarian%20cancer%20syndrome) | X |  |  |  |  |
| *BRCA2* | 794 | [Hereditary breast and ovarian cancer syndrome](https://www.orpha.net/consor/cgi-bin/Disease_Search.php?lng=EN&data_id=3384&MISSING%20CONTENT=Hereditary-breast-and-ovarian-cancer-syndrome&search=Disease_Search_Simple&title=Hereditary%20breast%20and%20ovarian%20cancer%20syndrome) | X |  |  |  |  |
| *BRIP1* | 519 | [Hereditary breast and ovarian cancer syndrome](https://www.orpha.net/consor/cgi-bin/Disease_Search.php?lng=EN&data_id=3384&MISSING%20CONTENT=Hereditary-breast-and-ovarian-cancer-syndrome&search=Disease_Search_Simple&title=Hereditary%20breast%20and%20ovarian%20cancer%20syndrome) | X |  |  |  |  |
| *BTD* | 224 | [Biotinidase deficiency](https://www.orpha.net/consor/cgi-bin/Disease_Search.php?lng=EN&data_id=11267&MISSING%20CONTENT=Biotinidase-deficiency&search=Disease_Search_Simple&title=Biotinidase%20deficiency) |  |  | X |  |  |
| *BTK* | 163 | [X-linked agammaglobulinemia](https://www.orpha.net/consor/cgi-bin/Disease_Search.php?lng=EN&data_id=142&MISSING%20CONTENT=X-linked-agammaglobulinemia&search=Disease_Search_Simple&title=X-linked%20agammaglobulinemia) |  | X |  |  |  |
| *C21orf2* | 78 | [Amyotrophic lateral sclerosis](https://www.orpha.net/consor/cgi-bin/Disease_Search.php?lng=EN&data_id=106&MISSING%20CONTENT=Amyotrophic-lateral-sclerosis&search=Disease_Search_Simple&title=Amyotrophic%20lateral%20sclerosis) |  |  |  | X |  |
| *CASP3* | 138 | NA |  |  |  |  | X |
| *CCM2* | 433 | [Familial cerebral cavernous malformation](https://www.orpha.net/consor/cgi-bin/Disease_Search.php?lng=EN&data_id=18935&MISSING%20CONTENT=Familial-cerebral-cavernous-malformation&search=Disease_Search_Simple&title=Familial%20cerebral%20cavernous%20malformation) |  | X |  |  |  |
| *CD300E* | 54 | NA |  |  |  |  | X |
| *CD40* | 49 | [X-linked hyper-IgM syndrome](https://www.orpha.net/consor/cgi-bin/Disease_Search.php?lng=EN&data_id=14799&MISSING%20CONTENT=X-linked-hyper-IgM-syndrome&search=Disease_Search_Simple&title=X-linked%20hyper-IgM%20syndrome) |  |  |  | X |  |
| *CD40LG* | 26 | [X-linked hyper-IgM syndrome](https://www.orpha.net/consor/cgi-bin/Disease_Search.php?lng=EN&data_id=14799&MISSING%20CONTENT=X-linked-hyper-IgM-syndrome&search=Disease_Search_Simple&title=X-linked%20hyper-IgM%20syndrome) |  |  |  | X |  |
| *CDH1* | 487 | [Hereditary diffuse gastric cancer](https://www.orpha.net/consor/cgi-bin/Disease_Search.php?lng=EN&data_id=8758&MISSING%20CONTENT=Hereditary-diffuse-gastric-cancer&search=Disease_Search_Simple&title=Hereditary%20diffuse%20gastric%20cancer) | X |  |  |  |  |
| *CDK5RAP2* | 491 | [Autosomal recessive primary microcephaly](https://www.orpha.net/consor/cgi-bin/Disease_Search.php?lng=EN&data_id=732&MISSING%20CONTENT=Autosomal-recessive-primary-microcephaly&search=Disease_Search_Simple&title=Autosomal%20recessive%20primary%20microcephaly) |  | X |  |  |  |
| *CDKN2A* | 18 | [Familial atypical multiple mole melanoma syndrome](https://www.orpha.net/consor/cgi-bin/Disease_Search.php?lng=EN&data_id=22939&MISSING%20CONTENT=Familial-atypical-multiple-mole-melanoma-syndrome&search=Disease_Search_Simple&title=Familial%20atypical%20multiple%20mole%20melanoma%20syndrome) | X |  |  |  |  |
| *CEP290* | 221 | [Bardet-Biedl syndrome](https://www.orpha.net/consor/cgi-bin/Disease_Search.php?lng=EN&data_id=3244&MISSING%20CONTENT=Bardet-Biedl-syndrome&search=Disease_Search_Simple&title=Bardet-Biedl%20syndrome) |  |  | X |  |  |
| *CFTR* | 693 | [Cystic fibrosis](https://www.orpha.net/consor/cgi-bin/Disease_Search.php?lng=EN&data_id=49&MISSING%20CONTENT=Cystic-fibrosis&search=Disease_Search_Simple&title=Cystic%20fibrosis) |  | X |  |  |  |
| *CHD7* | 620 | [CHARGE syndrome](https://www.orpha.net/consor/cgi-bin/Disease_Search.php?lng=EN&data_id=110&MISSING%20CONTENT=CHARGE-syndrome&search=Disease_Search_Simple&title=CHARGE%20syndrome) |  | X |  |  |  |
| *CHRNE* | 34 | [Postsynaptic congenital myasthenic syndromes](https://www.orpha.net/consor/cgi-bin/Disease_Search.php?lng=EN&data_id=13930&MISSING%20CONTENT=Postsynaptic-congenital-myasthenic-syndromes&search=Disease_Search_Simple&title=Postsynaptic%20congenital%20myasthenic%20syndromes) |  | X |  |  |  |
| *CISD2* | 91 | [Wolfram syndrome](https://www.orpha.net/consor/cgi-bin/Disease_Search.php?lng=EN&data_id=812&MISSING%20CONTENT=Wolfram-syndrome&search=Disease_Search_Simple&title=Wolfram%20syndrome) |  |  | X |  |  |
| *COL1A1* | 76 | [Arthrochalasia Ehlers-Danlos syndrome](https://www.orpha.net/consor/cgi-bin/Disease_Search.php?lng=EN&data_id=4044&MISSING%20CONTENT=Arthrochalasia-Ehlers-Danlos-syndrome&search=Disease_Search_Simple&title=Arthrochalasia%20Ehlers-Danlos%20syndrome) |  |  | X |  |  |
| *COL2A1* | 193 | [Hypochondrogenesis](https://www.orpha.net/consor/cgi-bin/Disease_Search.php?lng=EN&data_id=12234&MISSING%20CONTENT=Hypochondrogenesis&search=Disease_Search_Simple&title=Hypochondrogenesis) |  |  | X |  |  |
| *COL4A5* | 533 | [X-linked Alport syndrome](https://www.orpha.net/consor/cgi-bin/Disease_Search.php?lng=EN&data_id=11849&MISSING%20CONTENT=X-linked-Alport-syndrome&search=Disease_Search_Simple&title=X-linked%20Alport%20syndrome) |  |  | X |  |  |
| *COL5A1* | 1535 | [Classical Ehlers-Danlos syndrome](https://www.orpha.net/consor/cgi-bin/Disease_Search.php?lng=EN&data_id=612&MISSING%20CONTENT=Classical-Ehlers-Danlos-syndrome&search=Disease_Search_Simple&title=Classical%20Ehlers-Danlos%20syndrome) |  | X |  |  |  |
| *COL7A1* | 34 | [Autosomal dominant generalized dystrophic epidermolysis bullosa](https://www.orpha.net/consor/cgi-bin/Disease_Search.php?lng=EN&data_id=19201&MISSING%20CONTENT=Autosomal-dominant-generalized-dystrophic-epidermolysis-bullosa&search=Disease_Search_Simple&title=Autosomal%20dominant%20generalized%20dystrophic%20epidermolysis%20bullosa) |  | X |  |  |  |
| *CPOX* | 63 | [Hereditary coproporphyria](https://www.orpha.net/consor/cgi-bin/Disease_Search.php?lng=EN&data_id=11299&MISSING%20CONTENT=Hereditary-coproporphyria&search=Disease_Search_Simple&title=Hereditary%20coproporphyria) |  |  | X |  |  |
| *CPS1* | 406 | [Carbamoyl-phosphate synthetase 1 deficiency](https://www.orpha.net/consor/cgi-bin/Disease_Search.php?lng=EN&data_id=461&MISSING%20CONTENT=Carbamoyl-phosphate-synthetase-1-deficiency&search=Disease_Search_Simple&title=Carbamoyl-phosphate%20synthetase%201%20deficiency) |  |  | X |  |  |
| *CRISPLD2* | 657 | NA |  |  |  |  | X |
| *CTDP1* | 536 | [Congenital cataracts-facial dysmorphism-neuropathy syndrome](https://www.orpha.net/consor/cgi-bin/Disease_Search.php?lng=EN&data_id=10625&MISSING%20CONTENT=Congenital-cataracts-facial-dysmorphism-neuropathy-syndrome&search=Disease_Search_Simple&title=Congenital%20cataracts-facial%20dysmorphism-neuropathy%20syndrome) |  | X |  |  |  |
| *CTDSP1* | 34 | NA |  |  |  |  | X |
| *CTRC* | 40 | [Tropical pancreatitis](https://www.orpha.net/consor/cgi-bin/Disease_Search.php?lng=EN&data_id=14991&MISSING%20CONTENT=Tropical-pancreatitis&search=Disease_Search_Simple&title=Tropical%20pancreatitis) |  |  | X |  |  |
| *CUBN* | 1852 | [Imerslund-Gräsbeck syndrome](https://www.orpha.net/consor/cgi-bin/Disease_Search.php?lng=EN&data_id=10402&MISSING%20CONTENT=Imerslund-Grasbeck-syndrome&search=Disease_Search_Simple&title=Imerslund-Gräsbeck%20syndrome) |  |  | X |  |  |
| *CYBB* | 53 | [Chronic granulomatous disease](https://www.orpha.net/consor/cgi-bin/Disease_Search.php?lng=EN&data_id=176&MISSING%20CONTENT=Chronic-granulomatous-disease&search=Disease_Search_Simple&title=Chronic%20granulomatous%20disease) |  |  | X |  |  |
| *CYP27A1* | 120 | [Cerebrotendinous xanthomatosis](https://www.orpha.net/consor/cgi-bin/Disease_Search.php?lng=EN&data_id=605&MISSING%20CONTENT=Cerebrotendinous-xanthomatosis&search=Disease_Search_Simple&title=Cerebrotendinous%20xanthomatosis) |  |  | X |  |  |
| *DBT* | 193 | [Classic maple syrup urine disease](https://www.orpha.net/consor/cgi-bin/Disease_Search.php?lng=EN&data_id=20168&MISSING%20CONTENT=Classic-maple-syrup-urine-disease&search=Disease_Search_Simple&title=Classic%20maple%20syrup%20urine%20disease) |  | X |  |  |  |
| *DGUOK* | 155 | [Adult-onset multiple mitochondrial DNA deletion syndrome due to DGUOK deficiency](https://www.orpha.net/consor/cgi-bin/Disease_Search.php?lng=EN&data_id=21925&MISSING%20CONTENT=Adult-onset-multiple-mitochondrial-DNA-deletion-syndrome-due-to-DGUOK-deficiency&search=Disease_Search_Simple&title=Adult-onset%20multiple%20mitochondrial%20DNA%20deletion%20syndrome%20due%20to%20DGUOK%20deficiency) |  |  | X |  |  |
| *DHX36* | 150 | NA |  |  |  |  | X |
| *DISP2* | 38 | NA |  |  |  |  | X |
| *DMD* | 9083 | [Duchenne muscular dystrophy](https://www.orpha.net/consor/cgi-bin/Disease_Search.php?lng=EN&data_id=13913&MISSING%20CONTENT=Duchenne-muscular-dystrophy&search=Disease_Search_Simple&title=Duchenne%20muscular%20dystrophy) |  |  | X |  |  |
| *DNAH11* | 2521 | [Primary ciliary dyskinesia](https://www.orpha.net/consor/cgi-bin/Disease_Search.php?lng=EN&data_id=665&MISSING%20CONTENT=Primary-ciliary-dyskinesia&search=Disease_Search_Simple&title=Primary%20ciliary%20dyskinesia) |  | X |  |  |  |
| *DNAH5* | 1415 | [Primary ciliary dyskinesia](https://www.orpha.net/consor/cgi-bin/Disease_Search.php?lng=EN&data_id=665&MISSING%20CONTENT=Primary-ciliary-dyskinesia&search=Disease_Search_Simple&title=Primary%20ciliary%20dyskinesia) |  | X |  |  |  |
| *DNAJC13* | 483 | [Hereditary late-onset Parkinson disease](https://www.orpha.net/consor/cgi-bin/Disease_Search.php?lng=EN&data_id=23022&MISSING%20CONTENT=Hereditary-late-onset-Parkinson-disease&search=Disease_Search_Simple&title=Hereditary%20late-onset%20Parkinson%20disease) |  |  |  | X |  |
| *DYSF* | 1289 | [Congenital myopathy, Paradas type](https://www.orpha.net/consor/cgi-bin/Disease_Search.php?lng=EN&data_id=18402&MISSING%20CONTENT=Congenital-myopathy--Paradas-type&search=Disease_Search_Simple&title=Congenital%20myopathy,%20Paradas%20type) |  | X |  |  |  |
| *EED* | 199 | [Weaver syndrome](https://www.orpha.net/consor/cgi-bin/Disease_Search.php?lng=EN&data_id=604&MISSING%20CONTENT=Weaver-syndrome&search=Disease_Search_Simple&title=Weaver%20syndrome) |  | X |  |  |  |
| *EGFLAM* | 1171 | NA |  |  |  |  | X |
| *ELANE* | 16 | [Autosomal dominant severe congenital neutropenia](https://www.orpha.net/consor/cgi-bin/Disease_Search.php?lng=EN&data_id=822&MISSING%20CONTENT=Autosomal-dominant-severe-congenital-neutropenia&search=Disease_Search_Simple&title=Autosomal%20dominant%20severe%20congenital%20neutropenia) |  |  |  | X |  |
| *ELP1* | 382 | [Familial dysautonomia](https://www.orpha.net/consor/cgi-bin/Disease_Search.php?lng=EN&data_id=474&MISSING%20CONTENT=Familial-dysautonomia&search=Disease_Search_Simple&title=Familial%20dysautonomia) |  |  |  | X |  |
| *ENG* | 165 | [Hereditary hemorrhagic telangiectasia](https://www.orpha.net/consor/cgi-bin/Disease_Search.php?lng=EN&data_id=236&MISSING%20CONTENT=Hereditary-hemorrhagic-telangiectasia&search=Disease_Search_Simple&title=Hereditary%20hemorrhagic%20telangiectasia) |  |  |  | X |  |
| *EPB42* | 68 | [Hereditary spherocytosis](https://www.orpha.net/consor/cgi-bin/Disease_Search.php?lng=EN&data_id=3252&MISSING%20CONTENT=Hereditary-spherocytosis&search=Disease_Search_Simple&title=Hereditary%20spherocytosis) |  |  |  | X |  |
| *F8* | 215 | [Mild hemophilia A](https://www.orpha.net/consor/cgi-bin/Disease_Search.php?lng=EN&data_id=17874&MISSING%20CONTENT=Mild-hemophilia-A&search=Disease_Search_Simple&title=Mild%20hemophilia%20A) |  |  |  | X |  |
| *F9* | 90 | [Mild hemophilia B](https://www.orpha.net/consor/cgi-bin/Disease_Search.php?lng=EN&data_id=17871&MISSING%20CONTENT=Mild-hemophilia-B&search=Disease_Search_Simple&title=Mild%20hemophilia%20B) |  |  |  | X |  |
| *FAH* | 187 | [Tyrosinemia type 1](https://www.orpha.net/consor/cgi-bin/Disease_Search.php?lng=EN&data_id=3494&MISSING%20CONTENT=Tyrosinemia-type-1&search=Disease_Search_Simple&title=Tyrosinemia%20type%201) |  | X |  |  |  |
| *FANCA* | 864 | [Fanconi anemia](https://www.orpha.net/consor/cgi-bin/Disease_Search.php?lng=EN&data_id=634&MISSING%20CONTENT=Fanconi-anemia&search=Disease_Search_Simple&title=Fanconi%20anemia) | X |  |  |  |  |
| *FANCB* | 55 | [Fanconi anemia](https://www.orpha.net/consor/cgi-bin/Disease_Search.php?lng=EN&data_id=634&MISSING%20CONTENT=Fanconi-anemia&search=Disease_Search_Simple&title=Fanconi%20anemia) | X |  |  |  |  |
| *FANCC* | 623 | [Fanconi anemia](https://www.orpha.net/consor/cgi-bin/Disease_Search.php?lng=EN&data_id=634&MISSING%20CONTENT=Fanconi-anemia&search=Disease_Search_Simple&title=Fanconi%20anemia) | X |  |  |  |  |
| *FAS* | 327 | [Autoimmune lymphoproliferative syndrome](https://www.orpha.net/consor/cgi-bin/Disease_Search.php?lng=EN&data_id=3468&MISSING%20CONTENT=Autoimmune-lymphoproliferative-syndrome&search=Disease_Search_Simple&title=Autoimmune%20lymphoproliferative%20syndrome) | X |  |  |  |  |
| *FBN1* | 826 | [Acromicric dysplasia](https://www.orpha.net/consor/cgi-bin/Disease_Search.php?lng=EN&data_id=1289&MISSING%20CONTENT=Acromicric-dysplasia&search=Disease_Search_Simple&title=Acromicric%20dysplasia) | X |  |  |  |  |
| *FBN2* | 839 | [Congenital contractural arachnodactyly](https://www.orpha.net/consor/cgi-bin/Disease_Search.php?lng=EN&data_id=1481&MISSING%20CONTENT=Congenital-contractural-arachnodactyly&search=Disease_Search_Simple&title=Congenital%20contractural%20arachnodactyly) |  | X |  |  |  |
| *FECH* | 247 | [Autosomal erythropoietic protoporphyria](https://www.orpha.net/consor/cgi-bin/Disease_Search.php?lng=EN&data_id=11304&MISSING%20CONTENT=Autosomal-erythropoietic-protoporphyria&search=Disease_Search_Simple&title=Autosomal%20erythropoietic%20protoporphyria) |  |  | X |  |  |
| *FGB* | 47 | [Familial afibrinogenemia](https://www.orpha.net/consor/cgi-bin/Disease_Search.php?lng=EN&data_id=13897&MISSING%20CONTENT=Familial-afibrinogenemia&search=Disease_Search_Simple&title=Familial%20afibrinogenemia) |  |  | X |  |  |
| *FGD1* | 114 | [Aarskog-Scott syndrome](https://www.orpha.net/consor/cgi-bin/Disease_Search.php?lng=EN&data_id=394&MISSING%20CONTENT=Aarskog-Scott-syndrome&search=Disease_Search_Simple&title=Aarskog-Scott%20syndrome) | X |  |  |  |  |
| *FGG* | 13 | [Familial afibrinogenemia](https://www.orpha.net/consor/cgi-bin/Disease_Search.php?lng=EN&data_id=13897&MISSING%20CONTENT=Familial-afibrinogenemia&search=Disease_Search_Simple&title=Familial%20afibrinogenemia) |  | X |  |  |  |
| *FLNA* | 66 | [Congenital short bowel syndrome](https://www.orpha.net/consor/cgi-bin/Disease_Search.php?lng=EN&data_id=516&MISSING%20CONTENT=Congenital-short-bowel-syndrome&search=Disease_Search_Simple&title=Congenital%20short%20bowel%20syndrome) |  | X |  |  |  |
| *GAA* | 175 | [Glycogen storage disease due to acid maltase deficiency, infantile onset](https://www.orpha.net/consor/cgi-bin/Disease_Search.php?lng=EN&data_id=21321&MISSING%20CONTENT=Glycogen-storage-disease-due-to-acid-maltase-deficiency--infantile-onset&search=Disease_Search_Simple&title=Glycogen%20storage%20disease%20due%20to%20acid%20maltase%20deficiency,%20infantile%20onset) |  |  | X |  |  |
| *GALT* | 17 | [Classic galactosemia](https://www.orpha.net/consor/cgi-bin/Disease_Search.php?lng=EN&data_id=11265&MISSING%20CONTENT=Classic-galactosemia&search=Disease_Search_Simple&title=Classic%20galactosemia) |  |  | X |  |  |
| *GBA* | 23 | [Fetal Gaucher disease](https://www.orpha.net/consor/cgi-bin/Disease_Search.php?lng=EN&data_id=11662&MISSING%20CONTENT=Fetal-Gaucher-disease&search=Disease_Search_Simple&title=Fetal%20Gaucher%20disease) |  | X |  |  |  |
| *GH1* | 22 | [Isolated growth hormone deficiency type IA](https://www.orpha.net/consor/cgi-bin/Disease_Search.php?lng=EN&data_id=19209&MISSING%20CONTENT=Isolated-growth-hormone-deficiency-type-IA&search=Disease_Search_Simple&title=Isolated%20growth%20hormone%20deficiency%20type%20IA) |  | X |  |  |  |
| *GHR* | 1118 | [Laron syndrome](https://www.orpha.net/consor/cgi-bin/Disease_Search.php?lng=EN&data_id=3250&MISSING%20CONTENT=Laron-syndrome&search=Disease_Search_Simple&title=Laron%20syndrome) | X |  |  |  |  |
| *GLA* | 30 | [Fabry disease](https://www.orpha.net/consor/cgi-bin/Disease_Search.php?lng=EN&data_id=94&MISSING%20CONTENT=Fabry-disease&search=Disease_Search_Simple&title=Fabry%20disease) |  |  | X |  |  |
| *GNAT2* | 40 | [Achromatopsia](https://www.orpha.net/consor/cgi-bin/Disease_Search.php?lng=EN&data_id=10639&MISSING%20CONTENT=Achromatopsia&search=Disease_Search_Simple&title=Achromatopsia) |  | X |  |  |  |
| *GPAM* | 133 | NA |  |  |  |  | X |
| *GPR143* | 139 | [X-linked recessive ocular albinism](https://www.orpha.net/consor/cgi-bin/Disease_Search.php?lng=EN&data_id=629&MISSING%20CONTENT=X-linked-recessive-ocular-albinism&search=Disease_Search_Simple&title=X-linked%20recessive%20ocular%20albinism) |  | X |  |  |  |
| *GPT2* | 82 | [Postnatal MIHSDDID syndrome](https://www.orpha.net/consor/cgi-bin/Disease_Search.php?lng=EN&data_id=25138&MISSING%20CONTENT=Postnatal-microcephaly-infantile-hypotonia-spastic-diplegia-dysarthria-intellectual-disability-syndrome&search=Disease_Search_Simple&title=Postnatal%20microcephaly-infantile%20hypotonia-spastic%20diplegia-dysarthria-intellectual%20disability%20syndrome) |  | X |  |  |  |
| *GUSB* | 85 | [Mucopolysaccharidosis type 7](https://www.orpha.net/consor/cgi-bin/Disease_Search.php?lng=EN&data_id=40&MISSING%20CONTENT=Mucopolysaccharidosis-type-7&search=Disease_Search_Simple&title=Mucopolysaccharidosis%20type%207) |  |  | X |  |  |
| *HADHB* | 183 | [Mitochondrial trifunctional protein deficiency](https://www.orpha.net/consor/cgi-bin/Disease_Search.php?lng=EN&data_id=3294&MISSING%20CONTENT=Mitochondrial-trifunctional-protein-deficiency&search=Disease_Search_Simple&title=Mitochondrial%20trifunctional%20protein%20deficiency) |  |  | X |  |  |
| *HBA2* | 2 | [Hemoglobin H disease](https://www.orpha.net/consor/cgi-bin/Disease_Search.php?lng=EN&data_id=12449&MISSING%20CONTENT=Hemoglobin-H-disease&search=Disease_Search_Simple&title=Hemoglobin%20H%20disease) |  |  |  | X |  |
| *HBB* | 15 | [Beta-thalassemia intermedia](https://www.orpha.net/consor/cgi-bin/Disease_Search.php?lng=EN&data_id=19172&MISSING%20CONTENT=Beta-thalassemia-intermedia&search=Disease_Search_Simple&title=Beta-thalassemia%20intermedia) |  |  |  | X |  |
| *HCN2* | 267 | NA |  |  |  |  | X |
| *HEXA* | 144 | [Tay-Sachs disease, B1 variant](https://www.orpha.net/consor/cgi-bin/Disease_Search.php?lng=EN&data_id=21359&MISSING%20CONTENT=Tay-Sachs-disease--B1-variant&search=Disease_Search_Simple&title=Tay-Sachs%20disease,%20B1%20variant) |  |  | X |  |  |
| *HEXB* | 201 | [Sandhoff disease, adult form](https://www.orpha.net/consor/cgi-bin/Disease_Search.php?lng=EN&data_id=21355&MISSING%20CONTENT=Sandhoff-disease--adult-form&search=Disease_Search_Simple&title=Sandhoff%20disease,%20adult%20form) |  |  |  | X |  |
| *HFE* | 32 | [Symptomatic form of hemochromatosis type 1](https://www.orpha.net/consor/cgi-bin/Disease_Search.php?lng=EN&data_id=24056&MISSING%20CONTENT=Symptomatic-form-of-hemochromatosis-type-1&search=Disease_Search_Simple&title=Symptomatic%20form%20of%20hemochromatosis%20type%201) |  |  | X |  |  |
| *HFE2* | 1 | [Hemochromatosis type 2](https://www.orpha.net/consor/cgi-bin/Disease_Search.php?lng=EN&data_id=11256&MISSING%20CONTENT=Hemochromatosis-type-2&search=Disease_Search_Simple&title=Hemochromatosis%20type%202) |  |  | X |  |  |
| *HJV* | 16 | [Hemochromatosis type 2](https://www.orpha.net/consor/cgi-bin/Disease_Search.php?lng=EN&data_id=11256&MISSING%20CONTENT=Hemochromatosis-type-2&search=Disease_Search_Simple&title=Hemochromatosis%20type%202) |  |  | X |  |  |
| *HLA-A* | 297 | [Birdshot chorioretinopathy](https://www.orpha.net/consor/cgi-bin/Disease_Search.php?lng=EN&data_id=3404&MISSING%20CONTENT=Birdshot-chorioretinopathy&search=Disease_Search_Simple&title=Birdshot%20chorioretinopathy) |  | X |  |  |  |
| *HMBS* | 31 | [Acute intermittent porphyria](https://www.orpha.net/consor/cgi-bin/Disease_Search.php?lng=EN&data_id=11302&MISSING%20CONTENT=Acute-intermittent-porphyria&search=Disease_Search_Simple&title=Acute%20intermittent%20porphyria) |  |  | X |  |  |
| *HPRT1* | 195 | [Hypoxanthine guanine phosphoribosyltransferase partial deficiency](https://www.orpha.net/consor/cgi-bin/Disease_Search.php?lng=EN&data_id=11259&MISSING%20CONTENT=Hypoxanthine-guanine-phosphoribosyltransferase-partial-deficiency&search=Disease_Search_Simple&title=Hypoxanthine%20guanine%20phosphoribosyltransferase%20partial%20deficiency) |  |  | X |  |  |
| *HSD11B2* | 16 | [Apparent mineralocorticoid excess](https://www.orpha.net/consor/cgi-bin/Disease_Search.php?lng=EN&data_id=8740&MISSING%20CONTENT=Apparent-mineralocorticoid-excess&search=Disease_Search_Simple&title=Apparent%20mineralocorticoid%20excess) |  |  | X |  |  |
| *HSD17B10* | 3 | [HSD10 disease, atypical type](https://www.orpha.net/consor/cgi-bin/Disease_Search.php?lng=EN&data_id=11685&MISSING%20CONTENT=HSD10-disease--atypical-type&search=Disease_Search_Simple&title=HSD10%20disease,%20atypical%20type) |  |  |  | X |  |
| *HSPG2* | 576 | [Dyssegmental dysplasia, Silverman-Handmaker](https://www.orpha.net/consor/cgi-bin/Disease_Search.php?lng=EN&data_id=1839&MISSING%20CONTENT=Dyssegmental-dysplasia--Silverman-Handmaker-type&search=Disease_Search_Simple&title=Dyssegmental%20dysplasia,%20Silverman-Handmaker%20type) |  | X |  |  |  |
| *IDS* | 73 | [Mucopolysaccharidosis type 2, attenuated form](https://www.orpha.net/consor/cgi-bin/Disease_Search.php?lng=EN&data_id=18825&MISSING%20CONTENT=Mucopolysaccharidosis-type-2--attenuated-form&search=Disease_Search_Simple&title=Mucopolysaccharidosis%20type%202,%20attenuated%20form) |  |  | X |  |  |
| *IDUA* | 138 | [Hurler-Scheie syndrome](https://www.orpha.net/consor/cgi-bin/Disease_Search.php?lng=EN&data_id=12383&MISSING%20CONTENT=Hurler-Scheie-syndrome&search=Disease_Search_Simple&title=Hurler-Scheie%20syndrome) |  |  | X |  |  |
| *IGHMBP2* | 158 | [Charcot-Marie-Tooth disease type 2S](https://www.orpha.net/consor/cgi-bin/Disease_Search.php?lng=EN&data_id=23437&MISSING%20CONTENT=Charcot-Marie-Tooth-disease-type-2S&search=Disease_Search_Simple&title=Charcot-Marie-Tooth%20disease%20type%202S) |  |  |  | X |  |
| *IKBKAP* | 1 | [Familial dysautonomia](https://www.orpha.net/consor/cgi-bin/Disease_Search.php?lng=EN&data_id=474&MISSING%20CONTENT=Familial-dysautonomia&search=Disease_Search_Simple&title=Familial%20dysautonomia) |  |  |  | X |  |
| *IL2RG* | 8 | [Omenn syndrome](https://www.orpha.net/consor/cgi-bin/Disease_Search.php?lng=EN&data_id=10452&MISSING%20CONTENT=Omenn-syndrome&search=Disease_Search_Simple&title=Omenn%20syndrome) |  |  | X |  |  |
| *INPP4A* | 391 | NA |  |  |  |  | X |
| *ITGB2* | 269 | [Leukocyte adhesion deficiency type I](https://www.orpha.net/consor/cgi-bin/Disease_Search.php?lng=EN&data_id=14415&MISSING%20CONTENT=Leukocyte-adhesion-deficiency-type-I&search=Disease_Search_Simple&title=Leukocyte%20adhesion%20deficiency%20type%20I) |  |  |  | X |  |
| *ITGB3* | 323 | [Autosomal dominant macrothrombocytopenia](https://www.orpha.net/consor/cgi-bin/Disease_Search.php?lng=EN&data_id=17007&MISSING%20CONTENT=Autosomal-dominant-macrothrombocytopenia&search=Disease_Search_Simple&title=Autosomal%20dominant%20macrothrombocytopenia) |  |  |  | X |  |
| *ITGB4* | 140 | [Aplasia cutis congenita](https://www.orpha.net/consor/cgi-bin/Disease_Search.php?lng=EN&data_id=3198&MISSING%20CONTENT=Aplasia-cutis-congenita&search=Disease_Search_Simple&title=Aplasia%20cutis%20congenita) |  | X |  |  |  |
| *ITSN2* | 425 | NA |  |  |  |  | X |
| *IVD* | 80 | [Isovaleric acidemia](https://www.orpha.net/consor/cgi-bin/Disease_Search.php?lng=EN&data_id=399&MISSING%20CONTENT=Isovaleric-acidemia&search=Disease_Search_Simple&title=Isovaleric%20acidemia) |  |  | X |  |  |
| *JAK3* | 124 | [T-B+ severe combined immunodeficiency due to JAK3 deficiency](https://www.orpha.net/consor/cgi-bin/Disease_Search.php?lng=EN&data_id=10366&MISSING%20CONTENT=T-B--severe-combined-immunodeficiency-due-to-JAK3-deficiency&search=Disease_Search_Simple&title=T-B+%20severe%20combined%20immunodeficiency%20due%20to%20JAK3%20deficiency) |  |  |  | X |  |
| *KANSL2* | 129 | NA |  |  |  |  | X |
| *KCNH2* | 153 | [Romano-Ward syndrome](https://www.orpha.net/consor/cgi-bin/Disease_Search.php?lng=EN&data_id=14727&MISSING%20CONTENT=Romano-Ward-syndrome&search=Disease_Search_Simple&title=Romano-Ward%20syndrome) |  | X |  |  |  |
| *KIAA0922* | 1 | NA |  |  |  |  | X |
| *KRIT1* | 104 | [Familial cerebral cavernous malformation](https://www.orpha.net/consor/cgi-bin/Disease_Search.php?lng=EN&data_id=18935&MISSING%20CONTENT=Familial-cerebral-cavernous-malformation&search=Disease_Search_Simple&title=Familial%20cerebral%20cavernous%20malformation) |  | X |  |  |  |
| *L1CAM* | 27 | [MASA syndrome](https://www.orpha.net/consor/cgi-bin/Disease_Search.php?lng=EN&data_id=541&MISSING%20CONTENT=MASA-syndrome&search=Disease_Search_Simple&title=MASA%20syndrome) |  | X |  |  |  |
| *LAMB3* | 222 | [Hypoplastic amelogenesis imperfecta](https://www.orpha.net/consor/cgi-bin/Disease_Search.php?lng=EN&data_id=14604&MISSING%20CONTENT=Hypoplastic-amelogenesis-imperfecta&search=Disease_Search_Simple&title=Hypoplastic%20amelogenesis%20imperfecta) |  | X |  |  |  |
| *LAMC2* | 344 | [Intermediate generalized junctional epidermolysis bullosa](https://www.orpha.net/consor/cgi-bin/Disease_Search.php?lng=EN&data_id=11428&MISSING%20CONTENT=Intermediate-generalized-junctional-epidermolysis-bullosa&search=Disease_Search_Simple&title=Intermediate%20generalized%20junctional%20epidermolysis%20bullosa) |  | X |  |  |  |
| *LCAT* | 15 | [Fish-eye disease](https://www.orpha.net/consor/cgi-bin/Disease_Search.php?lng=EN&data_id=11318&MISSING%20CONTENT=Fish-eye-disease&search=Disease_Search_Simple&title=Fish-eye%20disease) |  | X |  |  |  |
| *LDLR* | 301 | [Homozygous familial hypercholesterolemia](https://www.orpha.net/consor/cgi-bin/Disease_Search.php?lng=EN&data_id=22639&MISSING%20CONTENT=Homozygous-familial-hypercholesterolemia&search=Disease_Search_Simple&title=Homozygous%20familial%20hypercholesterolemia) |  |  | X |  |  |
| *LHCGR* | 405 | [Leydig cell hypoplasia due to complete LH resistance](https://www.orpha.net/consor/cgi-bin/Disease_Search.php?lng=EN&data_id=12834&MISSING%20CONTENT=Leydig-cell-hypoplasia-due-to-complete-LH-resistance&search=Disease_Search_Simple&title=Leydig%20cell%20hypoplasia%20due%20to%20complete%20LH%20resistance) |  | X |  |  |  |
| *LIPC* | 911 | [Hyperlipidemia due to hepatic triacylglycerol lipase deficiency](https://www.orpha.net/consor/cgi-bin/Disease_Search.php?lng=EN&data_id=16994&MISSING%20CONTENT=Hyperlipidemia-due-to-hepatic-triacylglycerol-lipase-deficiency&search=Disease_Search_Simple&title=Hyperlipidemia%20due%20to%20hepatic%20triacylglycerol%20lipase%20deficiency) |  |  | X |  |  |
| *LMNA* | 146 | [Atypical Werner syndrome](https://www.orpha.net/consor/cgi-bin/Disease_Search.php?lng=EN&data_id=11500&MISSING%20CONTENT=Atypical-Werner-syndrome&search=Disease_Search_Simple&title=Atypical%20Werner%20syndrome) |  | X |  |  |  |
| *LMX1B* | 474 | [Nail-patella syndrome](https://www.orpha.net/consor/cgi-bin/Disease_Search.php?lng=EN&data_id=392&MISSING%20CONTENT=Nail-patella-syndrome&search=Disease_Search_Simple&title=Nail-patella%20syndrome) |  | X |  |  |  |
| *LRP5* | 652 | [Endosteal hyperostosis, Worth type](https://www.orpha.net/consor/cgi-bin/Disease_Search.php?lng=EN&data_id=2538&MISSING%20CONTENT=Endosteal-hyperostosis--Worth-type&search=Disease_Search_Simple&title=Endosteal%20hyperostosis,%20Worth%20type) |  |  | X |  |  |
| *MAPT* | 1049 | [Progressive non-fluent aphasia](https://www.orpha.net/consor/cgi-bin/Disease_Search.php?lng=EN&data_id=14643&MISSING%20CONTENT=Progressive-non-fluent-aphasia&search=Disease_Search_Simple&title=Progressive%20non-fluent%20aphasia) |  |  |  | X |  |
| *MCCC2* | 248 | [3-methylcrotonyl-CoA carboxylase deficiency](https://www.orpha.net/consor/cgi-bin/Disease_Search.php?lng=EN&data_id=3297&MISSING%20CONTENT=3-methylcrotonyl-CoA-carboxylase-deficiency&search=Disease_Search_Simple&title=3-methylcrotonyl-CoA%20carboxylase%20deficiency) |  |  | X |  |  |
| *MFAP2* | 31 | NA |  |  |  |  | X |
| *MFGE8* | 133 | NA |  |  |  |  | X |
| *MIP* | 19 | [Cerulean cataract](https://www.orpha.net/consor/cgi-bin/Disease_Search.php?lng=EN&data_id=14006&MISSING%20CONTENT=Cerulean-cataract&search=Disease_Search_Simple&title=Cerulean%20cataract) |  | X |  |  |  |
| *MLH1* | 545 | [Lynch syndrome](https://www.orpha.net/consor/cgi-bin/Disease_Search.php?lng=EN&data_id=3245&MISSING%20CONTENT=Lynch-syndrome&search=Disease_Search_Simple&title=Lynch%20syndrome) | X |  |  |  |  |
| *MLYCD* | 163 | [Malonic aciduria](https://www.orpha.net/consor/cgi-bin/Disease_Search.php?lng=EN&data_id=3295&MISSING%20CONTENT=Malonic-aciduria&search=Disease_Search_Simple&title=Malonic%20aciduria) |  |  | X |  |  |
| *MROH2B* | 461 | NA |  |  |  |  | X |
| *MRPS35* | 187 | NA |  |  |  |  | X |
| *MSH2* | 688 | [Lynch syndrome](https://www.orpha.net/consor/cgi-bin/Disease_Search.php?lng=EN&data_id=3245&MISSING%20CONTENT=Lynch-syndrome&search=Disease_Search_Simple&title=Lynch%20syndrome) | X |  |  |  |  |
| *MSH6* | 219 | [Lynch syndrome](https://www.orpha.net/consor/cgi-bin/Disease_Search.php?lng=EN&data_id=3245&MISSING%20CONTENT=Lynch-syndrome&search=Disease_Search_Simple&title=Lynch%20syndrome) | X |  |  |  |  |
| *MTR* | 544 | [Methylcobalamin deficiency type cblG](https://www.orpha.net/consor/cgi-bin/Disease_Search.php?lng=EN&data_id=3351&MISSING%20CONTENT=Methylcobalamin-deficiency-type-cblG&search=Disease_Search_Simple&title=Methylcobalamin%20deficiency%20type%20cblG) |  |  | X |  |  |
| *MTRR* | 165 | [Methylcobalamin deficiency type cblE](https://www.orpha.net/consor/cgi-bin/Disease_Search.php?lng=EN&data_id=2063&MISSING%20CONTENT=Methylcobalamin-deficiency-type-cblE&search=Disease_Search_Simple&title=Methylcobalamin%20deficiency%20type%20cblE) |  |  | X |  |  |
| *MUT* | 98 | NA |  |  |  |  | X |
| *MVK* | 102 | [Mevalonic aciduria](https://www.orpha.net/consor/cgi-bin/Disease_Search.php?lng=EN&data_id=403&MISSING%20CONTENT=Mevalonic-aciduria&search=Disease_Search_Simple&title=Mevalonic%20aciduria) |  |  | X |  |  |
| *MYO6* | 799 | [Autosomal dominant non-syndromic sensorineural deafness type DFNA](https://www.orpha.net/consor/cgi-bin/Disease_Search.php?lng=EN&data_id=12046&MISSING%20CONTENT=Autosomal-dominant-non-syndromic-sensorineural-deafness-type-DFNA&search=Disease_Search_Simple&title=Autosomal%20dominant%20non-syndromic%20sensorineural%20deafness%20type%252) |  |  |  | X |  |
| *NCAM2* | 3522 | NA |  |  |  |  | X |
| *NF1* | 1921 | [Neurofibromatosis-Noonan syndrome](https://www.orpha.net/consor/cgi-bin/Disease_Search.php?lng=EN&data_id=930&MISSING%20CONTENT=Neurofibromatosis-Noonan-syndrome&search=Disease_Search_Simple&title=Neurofibromatosis-Noonan%20syndrome) |  | X |  |  |  |
| *NF2* | 318 | [Neurofibromatosis type 2](https://www.orpha.net/consor/cgi-bin/Disease_Search.php?lng=EN&data_id=183&MISSING%20CONTENT=Neurofibromatosis-type-2&search=Disease_Search_Simple&title=Neurofibromatosis%20type%202) | X |  |  |  |  |
| *NOS1* | 906 | [Idiopathic achalasia](https://www.orpha.net/consor/cgi-bin/Disease_Search.php?lng=EN&data_id=302&MISSING%20CONTENT=Idiopathic-achalasia&search=Disease_Search_Simple&title=Idiopathic%20achalasia) |  | X |  |  |  |
| *NPC1* | 194 | [Niemann-Pick disease type C, adult neurologic onset](https://www.orpha.net/consor/cgi-bin/Disease_Search.php?lng=EN&data_id=18805&MISSING%20CONTENT=Niemann-Pick-disease-type-C--adult-neurologic-onset&search=Disease_Search_Simple&title=Niemann-Pick%20disease%20type%20C,%20adult%20neurologic%20onset) |  |  | X |  |  |
| *NTRK1* | 87 | [Familial medullary thyroid carcinoma](https://www.orpha.net/consor/cgi-bin/Disease_Search.php?lng=EN&data_id=14205&MISSING%20CONTENT=Familial-medullary-thyroid-carcinoma&search=Disease_Search_Simple&title=Familial%20medullary%20thyroid%20carcinoma) | X |  |  |  |  |
| *OAT* | 262 | [Gyrate atrophy of choroid and retina](https://www.orpha.net/consor/cgi-bin/Disease_Search.php?lng=EN&data_id=3349&MISSING%20CONTENT=Gyrate-atrophy-of-choroid-and-retina&search=Disease_Search_Simple&title=Gyrate%20atrophy%20of%20choroid%20and%20retina) |  | X |  |  |  |
| *OCA2* | 1962 | [Oculocutaneous albinism type 2](https://www.orpha.net/consor/cgi-bin/Disease_Search.php?lng=EN&data_id=11458&MISSING%20CONTENT=Oculocutaneous-albinism-type-2&search=Disease_Search_Simple&title=Oculocutaneous%20albinism%20type%202) |  | X |  |  |  |
| *OXCT1* | 249 | [Succinyl-CoA:3-oxoacid CoA transferase deficiency](https://www.orpha.net/consor/cgi-bin/Disease_Search.php?lng=EN&data_id=3298&MISSING%20CONTENT=Succinyl-CoA-3-oxoacid-CoA-transferase-deficiency&search=Disease_Search_Simple&title=Succinyl-CoA:3-oxoacid%20CoA%20transferase%20deficiency) |  |  | X |  |  |
| *PALB2* | 95 | [Fanconi anemia](https://www.orpha.net/consor/cgi-bin/Disease_Search.php?lng=EN&data_id=634&MISSING%20CONTENT=Fanconi-anemia&search=Disease_Search_Simple&title=Fanconi%20anemia) | X |  |  |  |  |
| *PCCA* | 1791 | [Propionic acidemia](https://www.orpha.net/consor/cgi-bin/Disease_Search.php?lng=EN&data_id=3557&MISSING%20CONTENT=Propionic-acidemia&search=Disease_Search_Simple&title=Propionic%20acidemia) |  |  | X |  |  |
| *PCCB* | 342 | [Propionic acidemia](https://www.orpha.net/consor/cgi-bin/Disease_Search.php?lng=EN&data_id=3557&MISSING%20CONTENT=Propionic-acidemia&search=Disease_Search_Simple&title=Propionic%20acidemia) |  |  | X |  |  |
| *PDHA1* | 94 | [Leigh syndrome with leukodystrophy](https://www.orpha.net/consor/cgi-bin/Disease_Search.php?lng=EN&data_id=19814&MISSING%20CONTENT=Leigh-syndrome-with-leukodystrophy&search=Disease_Search_Simple&title=Leigh%20syndrome%20with%20leukodystrophy) |  | X |  |  |  |
| *PDK2* | 1 | NA |  |  |  |  | X |
| *PFKM* | 104 | [Glycogen storage disease due to muscle phosphofructokinase deficiency](https://www.orpha.net/consor/cgi-bin/Disease_Search.php?lng=EN&data_id=19&MISSING%20CONTENT=Glycogen-storage-disease-due-to-muscle-phosphofructokinase-deficiency&search=Disease_Search_Simple&title=Glycogen%20storage%20disease%20due%20to%20muscle%20phosphofructokinase%20deficiency) |  |  | X |  |  |
| *PHEX* | 781 | [X-linked hypophosphatemia](https://www.orpha.net/consor/cgi-bin/Disease_Search.php?lng=EN&data_id=11911&MISSING%20CONTENT=X-linked-hypophosphatemia&search=Disease_Search_Simple&title=X-linked%20hypophosphatemia) |  |  | X |  |  |
| *PKD1* | 464 | [Autosomal dominant polycystic kidney disease](https://www.orpha.net/consor/cgi-bin/Disease_Search.php?lng=EN&data_id=823&MISSING%20CONTENT=Autosomal-dominant-polycystic-kidney-disease&search=Disease_Search_Simple&title=Autosomal%20dominant%20polycystic%20kidney%20disease) |  |  | X |  |  |
| *PKD2* | 227 | [Autosomal dominant polycystic kidney disease](https://www.orpha.net/consor/cgi-bin/Disease_Search.php?lng=EN&data_id=823&MISSING%20CONTENT=Autosomal-dominant-polycystic-kidney-disease&search=Disease_Search_Simple&title=Autosomal%20dominant%20polycystic%20kidney%20disease) |  |  | X |  |  |
| *PKHD1* | 2363 | [Caroli disease](https://www.orpha.net/consor/cgi-bin/Disease_Search.php?lng=EN&data_id=10715&MISSING%20CONTENT=Caroli-disease&search=Disease_Search_Simple&title=Caroli%20disease) |  |  | X |  |  |
| *PLA2R1* | 666 | NA |  |  |  |  | X |
| *PLP1* | 28 | [Pelizaeus-Merzbacher disease, classic form](https://www.orpha.net/consor/cgi-bin/Disease_Search.php?lng=EN&data_id=20439&MISSING%20CONTENT=Pelizaeus-Merzbacher-disease--classic-form&search=Disease_Search_Simple&title=Pelizaeus-Merzbacher%20disease,%20classic%20form) |  |  |  | X |  |
| *PMM2* | 479 | [PMM2-CDG](https://www.orpha.net/consor/cgi-bin/Disease_Search.php?lng=EN&data_id=11344&MISSING%20CONTENT=PMM2-CDG&search=Disease_Search_Simple&title=PMM2-CDG) |  |  | X |  |  |
| *PMS2* | 251 | [Lynch syndrome](https://www.orpha.net/consor/cgi-bin/Disease_Search.php?lng=EN&data_id=3245&MISSING%20CONTENT=Lynch-syndrome&search=Disease_Search_Simple&title=Lynch%20syndrome) | X |  |  |  |  |
| *PRPF19* | 48 | NA |  |  |  |  | X |
| *PRPF31* | 88 | [Retinitis pigmentosa](https://www.orpha.net/consor/cgi-bin/Disease_Search.php?lng=EN&data_id=659&MISSING%20CONTENT=Retinitis-pigmentosa&search=Disease_Search_Simple&title=Retinitis%20pigmentosa) |  | X |  |  |  |
| *PTEN* | 264 | [Cowden syndrome](https://www.orpha.net/consor/cgi-bin/Disease_Search.php?lng=EN&data_id=243&MISSING%20CONTENT=Cowden-syndrome&search=Disease_Search_Simple&title=Cowden%20syndrome) | X |  |  |  |  |
| *PTH1R* | 78 | [Eiken syndrome](https://www.orpha.net/consor/cgi-bin/Disease_Search.php?lng=EN&data_id=11148&MISSING%20CONTENT=Eiken-syndrome&search=Disease_Search_Simple&title=Eiken%20syndrome) |  |  | X |  |  |
| *PTPN13* | 822 | NA |  |  |  |  | X |
| *PTPRC* | 457 | [T-B+ severe combined immunodeficiency due to CD45 deficiency](https://www.orpha.net/consor/cgi-bin/Disease_Search.php?lng=EN&data_id=17830&MISSING%20CONTENT=T-B--severe-combined-immunodeficiency-due-to-CD45-deficiency&search=Disease_Search_Simple&title=T-B+%20severe%20combined%20immunodeficiency%20due%20to%20CD45%20deficiency) |  |  |  | X |  |
| *RAD51C* | 125 | [Hereditary breast and ovarian cancer syndrome](https://www.orpha.net/consor/cgi-bin/Disease_Search.php?lng=EN&data_id=3384&MISSING%20CONTENT=Hereditary-breast-and-ovarian-cancer-syndrome&search=Disease_Search_Simple&title=Hereditary%20breast%20and%20ovarian%20cancer%20syndrome) | X |  |  |  |  |
| *RAD51D* | 65 | [Hereditary breast and ovarian cancer syndrome](https://www.orpha.net/consor/cgi-bin/Disease_Search.php?lng=EN&data_id=3384&MISSING%20CONTENT=Hereditary-breast-and-ovarian-cancer-syndrome&search=Disease_Search_Simple&title=Hereditary%20breast%20and%20ovarian%20cancer%20syndrome) | X |  |  |  |  |
| *RB1* | 557 | [Hereditary retinoblastoma](https://www.orpha.net/consor/cgi-bin/Disease_Search.php?lng=EN&data_id=22197&MISSING%20CONTENT=Hereditary-retinoblastoma&search=Disease_Search_Simple&title=Hereditary%20retinoblastoma) | X |  |  |  |  |
| *RBM23* | 102 | NA |  |  |  |  | X |
| *RET* | 302 | [Familial medullary thyroid carcinoma](https://www.orpha.net/consor/cgi-bin/Disease_Search.php?lng=EN&data_id=14205&MISSING%20CONTENT=Familial-medullary-thyroid-carcinoma&search=Disease_Search_Simple&title=Familial%20medullary%20thyroid%20carcinoma) | X |  |  |  |  |
| *RHD* | 318 | [Rh deficiency syndrome](https://www.orpha.net/consor/cgi-bin/Disease_Search.php?lng=EN&data_id=10997&MISSING%20CONTENT=Rh-deficiency-syndrome&search=Disease_Search_Simple&title=Rh%20deficiency%20syndrome) |  |  |  | X |  |
| *RYR1* | 880 | [Autosomal recessive centronuclear myopathy](https://www.orpha.net/consor/cgi-bin/Disease_Search.php?lng=EN&data_id=17833&MISSING%20CONTENT=Autosomal-recessive-centronuclear-myopathy&search=Disease_Search_Simple&title=Autosomal%20recessive%20centronuclear%20myopathy) |  |  | X |  |  |
| *SDK2* | 1562 | NA |  |  |  |  | X |
| *SEC14L3* | 75 | NA |  |  |  |  | X |
| *SEC31B* | 68 | NA |  |  |  |  | X |
| *SERPINC1* | 45 | [Hereditary thrombophilia due to congenital antithrombin deficiency](https://www.orpha.net/consor/cgi-bin/Disease_Search.php?lng=EN&data_id=3590&MISSING%20CONTENT=Hereditary-thrombophilia-due-to-congenital-antithrombin-deficiency&search=Disease_Search_Simple&title=Hereditary%20thrombophilia%20due%20to%20congenital%20antithrombin%20deficiency) |  |  |  | X |  |
| *SERPING1* | 79 | [Hereditary angioedema type 1](https://www.orpha.net/consor/cgi-bin/Disease_Search.php?lng=EN&data_id=14623&MISSING%20CONTENT=Hereditary-angioedema-type-1&search=Disease_Search_Simple&title=Hereditary%20angioedema%20type%201) |  | X |  |  |  |
| *SLC12A3* | 388 | [Gitelman syndrome](https://www.orpha.net/consor/cgi-bin/Disease_Search.php?lng=EN&data_id=1045&MISSING%20CONTENT=Gitelman-syndrome&search=Disease_Search_Simple&title=Gitelman%20syndrome) |  |  | X |  |  |
| *SLC26A4* | 166 | [Pendred syndrome](https://www.orpha.net/consor/cgi-bin/Disease_Search.php?lng=EN&data_id=558&MISSING%20CONTENT=Pendred-syndrome&search=Disease_Search_Simple&title=Pendred%20syndrome) |  |  |  | X |  |
| *SLC2A9* | 1334 | [Hereditary renal hypouricemia](https://www.orpha.net/consor/cgi-bin/Disease_Search.php?lng=EN&data_id=12557&MISSING%20CONTENT=Hereditary-renal-hypouricemia&search=Disease_Search_Simple&title=Hereditary%20renal%20hypouricemia) |  | X |  |  |  |
| *SLC40A1* | 107 | [Hemochromatosis type 4](https://www.orpha.net/consor/cgi-bin/Disease_Search.php?lng=EN&data_id=16909&MISSING%20CONTENT=Hemochromatosis-type-4&search=Disease_Search_Simple&title=Hemochromatosis%20type%204) |  |  |  | X |  |
| *SLC4A11* | 76 | [Congenital hereditary endothelial dystrophy type II](https://www.orpha.net/consor/cgi-bin/Disease_Search.php?lng=EN&data_id=20862&MISSING%20CONTENT=Congenital-hereditary-endothelial-dystrophy-type-II&search=Disease_Search_Simple&title=Congenital%20hereditary%20endothelial%20dystrophy%20type%20II) |  | X |  |  |  |
| *SLC5A5* | 142 | [Familial thyroid dyshormonogenesis](https://www.orpha.net/consor/cgi-bin/Disease_Search.php?lng=EN&data_id=12671&MISSING%20CONTENT=Familial-thyroid-dyshormonogenesis&search=Disease_Search_Simple&title=Familial%20thyroid%20dyshormonogenesis) |  |  | X |  |  |
| *SLC9C1* | 1019 | NA |  |  |  |  | X |
| *SMARCB1* | 483 | [Coffin-Siris syndrome](https://www.orpha.net/consor/cgi-bin/Disease_Search.php?lng=EN&data_id=321&MISSING%20CONTENT=Coffin-Siris-syndrome&search=Disease_Search_Simple&title=Coffin-Siris%20syndrome) |  |  |  | X |  |
| *SMN1* | 43 | [Proximal spinal muscular atrophy type 1](https://www.orpha.net/consor/cgi-bin/Disease_Search.php?lng=EN&data_id=11563&MISSING%20CONTENT=Proximal-spinal-muscular-atrophy-type-1&search=Disease_Search_Simple&title=Proximal%20spinal%20muscular%20atrophy%20type%201) |  |  |  | X |  |
| *SMN2* | 52 | [Proximal spinal muscular atrophy type 1](https://www.orpha.net/consor/cgi-bin/Disease_Search.php?lng=EN&data_id=11563&MISSING%20CONTENT=Proximal-spinal-muscular-atrophy-type-1&search=Disease_Search_Simple&title=Proximal%20spinal%20muscular%20atrophy%20type%201) |  |  |  | X |  |
| *SOD1* | 26 | [Amyotrophic lateral sclerosis](https://www.orpha.net/consor/cgi-bin/Disease_Search.php?lng=EN&data_id=106&MISSING%20CONTENT=Amyotrophic-lateral-sclerosis&search=Disease_Search_Simple&title=Amyotrophic%20lateral%20sclerosis) |  |  |  | X |  |
| *SPATS2L* | 659 | NA |  |  |  |  | X |
| *SPINK5* | 611 | [Netherton syndrome](https://www.orpha.net/consor/cgi-bin/Disease_Search.php?lng=EN&data_id=938&MISSING%20CONTENT=Netherton-syndrome&search=Disease_Search_Simple&title=Netherton%20syndrome) |  | X |  |  |  |
| *SPTA1* | 357 | [Hereditary elliptocytosis](https://www.orpha.net/consor/cgi-bin/Disease_Search.php?lng=EN&data_id=3655&MISSING%20CONTENT=Hereditary-elliptocytosis&search=Disease_Search_Simple&title=Hereditary%20elliptocytosis) |  |  |  | X |  |
| *SPTBN5* | 205 | NA |  |  |  |  | X |
| *STAT3* | 318 | [Autosomal dominant hyper-IgE syndrome](https://www.orpha.net/consor/cgi-bin/Disease_Search.php?lng=EN&data_id=839&MISSING%20CONTENT=Autosomal-dominant-hyper-IgE-syndrome&search=Disease_Search_Simple&title=Autosomal%20dominant%20hyper-IgE%20syndrome) |  |  |  | X |  |
| *SYTL1* | 26 | NA |  |  |  |  | X |
| *TAZ* | 18 | [Epithelioid hemangioendothelioma](https://www.orpha.net/consor/cgi-bin/Disease_Search.php?lng=EN&data_id=17144&MISSING%20CONTENT=Epithelioid-hemangioendothelioma&search=Disease_Search_Simple&title=Epithelioid%20hemangioendothelioma) |  | X |  |  |  |
| *TCIRG1* | 25 | [Autosomal dominant severe congenital neutropenia](https://www.orpha.net/consor/cgi-bin/Disease_Search.php?lng=EN&data_id=822&MISSING%20CONTENT=Autosomal-dominant-severe-congenital-neutropenia&search=Disease_Search_Simple&title=Autosomal%20dominant%20severe%20congenital%20neutropenia) |  |  | X |  |  |
| *TFR2* | 71 | [Hemochromatosis type 3](https://www.orpha.net/consor/cgi-bin/Disease_Search.php?lng=EN&data_id=18976&MISSING%20CONTENT=Hemochromatosis-type-3&search=Disease_Search_Simple&title=Hemochromatosis%20type%203) |  |  | X |  |  |
| *TH* | 50 | [Autosomal recessive dopa-responsive dystonia](https://www.orpha.net/consor/cgi-bin/Disease_Search.php?lng=EN&data_id=14826&MISSING%20CONTENT=Autosomal-recessive-dopa-responsive-dystonia&search=Disease_Search_Simple&title=Autosomal%20recessive%20dopa-responsive%20dystonia) |  |  | X |  |  |
| *TLE1* | 611 | NA |  |  |  |  | X |
| *TMEM131L* | 922 | NA |  |  |  |  | X |
| *TNFRSF1A* | 48 | [Tumor necrosis factor receptor 1 associated periodic syndrome](https://www.orpha.net/consor/cgi-bin/Disease_Search.php?lng=EN&data_id=10303&MISSING%20CONTENT=Tumor-necrosis-factor-receptor-1-associated-periodic-syndrome&search=Disease_Search_Simple&title=Tumor%20necrosis%20factor%20receptor%201%20associated%20periodic%20syndrome) |  |  |  | X |  |
| *TSC2* | 189 | [Tuberous sclerosis complex](https://www.orpha.net/consor/cgi-bin/Disease_Search.php?lng=EN&data_id=660&MISSING%20CONTENT=Tuberous-sclerosis-complex&search=Disease_Search_Simple&title=Tuberous%20sclerosis%20complex) |  |  |  | X |  |
| *TXNRD1* | 263 | NA |  |  |  |  | X |
| *UNC5C* | 1982 | NA |  |  |  |  | X |
| *UNC5CL* | 26 | NA |  |  |  |  | X |
| *UROS* | 126 | [Congenital erythropoietic porphyria](https://www.orpha.net/consor/cgi-bin/Disease_Search.php?lng=EN&data_id=11303&MISSING%20CONTENT=Congenital-erythropoietic-porphyria&search=Disease_Search_Simple&title=Congenital%20erythropoietic%20porphyria) |  |  |  | X |  |
| *USH2A* | 3360 | [Retinitis pigmentosa](https://www.orpha.net/consor/cgi-bin/Disease_Search.php?lng=EN&data_id=659&MISSING%20CONTENT=Retinitis-pigmentosa&search=Disease_Search_Simple&title=Retinitis%20pigmentosa) |  | X |  |  |  |
| *VDR* | 323 | [Hypocalcemic vitamin D-resistant rickets](https://www.orpha.net/consor/cgi-bin/Disease_Search.php?lng=EN&data_id=12173&MISSING%20CONTENT=Hypocalcemic-vitamin-D-resistant-rickets&search=Disease_Search_Simple&title=Hypocalcemic%20vitamin%20D-resistant%20rickets) |  |  | X |  |  |
| *VIPR2* | 704 | NA |  |  |  |  | X |
| *VWF* | 1119 | [Von Willebrand disease type 1](https://www.orpha.net/consor/cgi-bin/Disease_Search.php?lng=EN&data_id=17611&MISSING%20CONTENT=Von-Willebrand-disease-type-1&search=Disease_Search_Simple&title=Von%20Willebrand%20disease%20type%201) |  |  |  | X |  |
| *WAS* | 6 | [Wiskott-Aldrich syndrome](https://www.orpha.net/consor/cgi-bin/Disease_Search.php?lng=EN&data_id=144&MISSING%20CONTENT=Wiskott-Aldrich-syndrome&search=Disease_Search_Simple&title=Wiskott-Aldrich%20syndrome) |  |  |  | X |  |
| *WT1* | 430 | [Denys-Drash syndrome](https://www.orpha.net/consor/cgi-bin/Disease_Search.php?lng=EN&data_id=3552&MISSING%20CONTENT=Denys-Drash-syndrome&search=Disease_Search_Simple&title=Denys-Drash%20syndrome) |  |  | X |  |  |
| *XPA* | 109 | [Xeroderma pigmentosum](https://www.orpha.net/consor/cgi-bin/Disease_Search.php?lng=EN&data_id=3253&MISSING%20CONTENT=Xeroderma-pigmentosum&search=Disease_Search_Simple&title=Xeroderma%20pigmentosum) |  | X |  |  |  |
| *XPC* | 119 | [Xeroderma pigmentosum](https://www.orpha.net/consor/cgi-bin/Disease_Search.php?lng=EN&data_id=3253&MISSING%20CONTENT=Xeroderma-pigmentosum&search=Disease_Search_Simple&title=Xeroderma%20pigmentosum) |  | X |  |  |  |
| *ZDHHC6* | 62 | NA |  |  |  |  | X |

### **Supplementary Table S4:** Parameters of metascore model

| Parameters | Value | Standard error | P_Wald test_ |
| --- | --- | --- | --- |
| $\beta_{0}$ | -1.175e+01 | 3.485e-02 | 2e-16 |
| $\beta_{SSF-like}$ | 5.719e-02 | 4.652e-04 | 2e-16 |
| $\beta_{MES}$ | 4.541e-01 | 1.076e-03 | 2e-16 |
| $\beta_{ESR}$ | 7.005e+00 | 1.684e-02 | 2e-16 |
| $\beta_{sstype}$ | 1.789e+00 | 4.836e-02 | 2e-16 |
| $\beta_{sstype:SSF-like}$ | -1.825e-02 | 6.967e-04 | 2e-16 |
| $\beta_{sstype:MES}$ | 6.209e-02 | 1.965e-03 | 2e-16 |
| $\beta_{sstype:ESR}$ | 1.367e-01 | 2.425e-02 | 1.72e-08 |

### **Supplementary Table S5:** Selection of predictors for Random Forest model of SPiP. In bold predictor used in the final model. Qual.: Qualitative variable; Quan.: Quantitative variable; Bimo.: Bimodal variable.

| Predictor | Description | Type | Range | AUC  Average [Min ; Max] | p-value |
| --- | --- | --- | --- | --- | --- |
| ALL | All variables | - | - | 0,986 [0,98 ; 0,992] | 1 |
| nearestSStoCrypt | Splice site type of nearest natural splice site to new site | Qual. | (Donor; Acceptor) | 0,986 [0,979 ; 0,991] | 0,310 |
| sstypeCryptMut | Splice site type of new site | Qual. | (Donor; Acceptor) | 0,986 [0,979 ; 0,992] | 0,799 |
| NearestSS | Splice site type of nearest natural splice site to variant | Qual. | (Donor; Acceptor) | 0,985 [0,977 ; 0,99] | 0,075 |
| varType | Type of variation | Qual. | [substitution, delins, … ] | 0,984 [0,978 ; 0,988] | 0,004 |
| **BP** | **If variant is in BPP predicted branch point** | **Bimo.** | (0;1) | **0,982 [0,975 ; 0,988]** | **7,90E-08** |
| **deltaMES** | **Variation of MES score between wild-type and mutated sequence** | **Quan.** | [-∞;+∞] | **0,98 [0,973 ; 0,987]** | **3,46E-05** |
| probaSSPhysioMut | Score^†^ of the nearest natural splice site on mutated sequence | Quan. | [0;1] | 0,981 [0,973 ; 0,988] | 0,630 |
| nearestDistSStoCrypt | Relative distance between new site and natural splice site | Quan. | [-∞;+∞] | 0,98 [0,971 ; 0,986] | 0,188 |
| **probaCryptWT** | **Score^†^ of *de novo*/cryptic model on wild-type sequence** | **Quan.** | [0;1] | **0,977 [0,967 ; 0,985]** | **1,94E-11** |
| RegType | Type of pre-mRNA regions impacted by the variant | Qual. | (exon/intron) | 0,977 [0,967 ; 0,986] | 0,487 |
| probaSSPhysio | Score^†^ of the nearest natural splice site | Quan. | [0;1] | 0,976 [0,969 ; 0,983] | 0,021 |
| **probaCryptMut** | **Score^†^ of *de novo*/cryptic model on mutated sequence** | **Quan.** | [0;1] | **0,963 [0,953 ; 0,976]** | **1,42E-52** |
| **DistSS** | **Relative distance between variant and the nearest splice site** | **Quan.** | [-∞;+∞] | **0,936 [0,921 ; 0,952]** | **8,02E-90** |
| **exonSize** | **Size of exon/intron** | **Qual.** | [1;+∞] | **0,888 [0,866 ; 0,908]** | **6,8E-112** |
| **deltaESRscore** | **QUEPASA score** | **Quan.** | [-∞;+∞] | **0,775 [0,754 ; 0,799]** | **3,8E-177** |
| **SPiCEproba** | **SPiCE score** | **Quan.** | [0;1] | **0** | **0** |

^†^Scores given by the new metascore to predict splice site creation or reinforcement

### **Supplementary Table S6:** Selection of optimal number of predictors sampled at each node (mtry). *kept number

| mtry | AUC ROC |
| --- | --- |
| 1 | 0.966 |
| 2 | 0.976 |
| 3* | 0.986 |
| 4 | 0.983 |
| 5 | 0.981 |
| 6 | 0.983 |
| 7 | 0.977 |
| 8 | 0.987 |
| 9 | 0.977 |

### **Supplementary Table S7:** SPiP efficiency on SpliceAI missing data, n = 914 variants.

|  | SPiP, n = 914 |
| --- | --- |
| True positives | 45 |
| False positives | 58 |
| True negatives | 807 |
| False negatives | 4 |
| Accuracy | 93.22 % |
| Sensitivity | 91.84 % |
| Specificity | 93.29 % |

### **Supplementary Table S8:** Performance of SPiP versus SpliceAI and SQUIRLS on variants without the ‘controls’ (n = 2,320 variants). Chi-square test ‘*’: p-value < 0.05; ‘**’: p-value < 0.01; ‘***’: p-value < 0.001. AUC: Area Under the Curve (ROC curve), PR_AUC: Area Under the Curve of Precision Recall curve.

|  | SPiP  Average  [min ; max] | SpliceAI  Average  [min ; max] | SQUIRLS  Average  [min ; max] |
| --- | --- | --- | --- |
| AUC | 0.921***  [0.904 ; 0.934] | 0.901  [0.886 ; 0.914] | 0.835  [0.815 ; 0.864] |
| PR_AUC | 0.920***  [0.907 ; 0.932] | 0.902  [0.886 ; 0.916] | 0.848  [0.828 ; 0.876] |

## **SUPPLEMENTARY FIGURES**


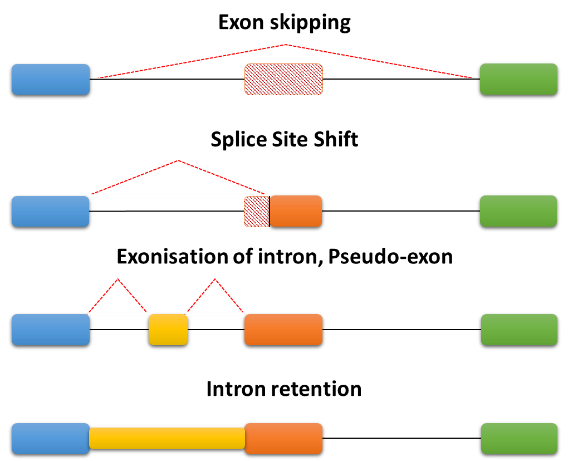


### **Supplementary** **Figure S1:** Illustration of variant-induced splicing alterations.


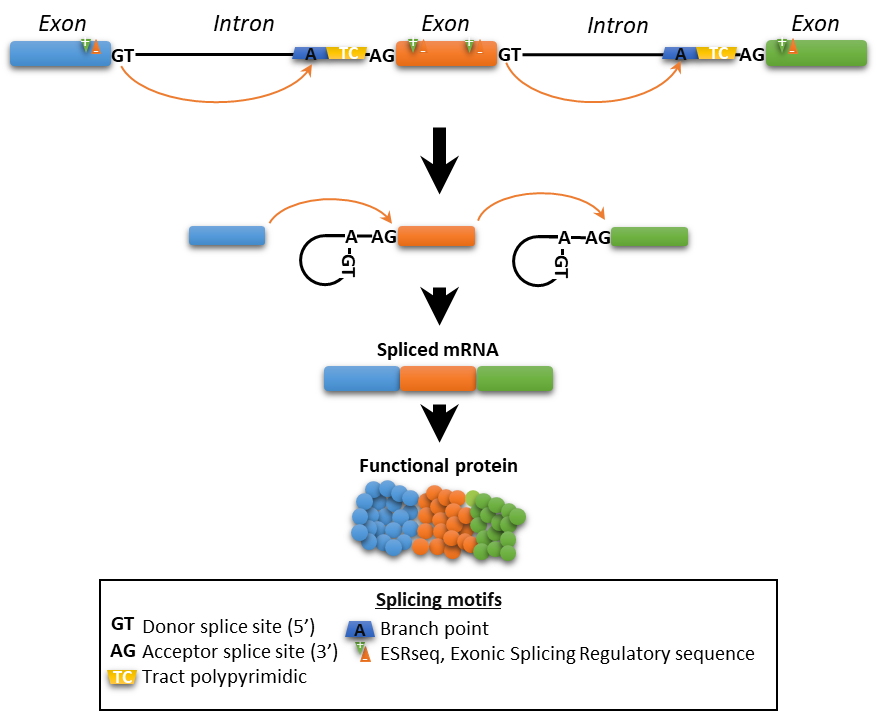


### **Supplementary** **Figure S2:** Splicing mechanism and motifs.

The donor splice site defines the exon/intron junction, with a highly conserved intronic dinucleotide (GT). The acceptor splice site delineates the intron/exon junction, with a highly conserved dinucleotide (AG). The branch site is a short motif upstream the 3’ss that includes the branch point (BP) adenosine. These BPs are mainly located in the area between -44 and -18 nucleotide of the natural 3’ss (Mercer et al., 2015). Separating the 3’ss and the BPs area, there is a cytosine and thymidine rich sequence called polypyrimidine tract (PPT). Splicing efficiency also depends of short auxiliary motifs (6-8 nt) defined as splicing regulatory elements (SREs). Briefly, these motifs are binding signals for RNA-binding proteins, mostly SR (serine and arginine rich) and HnRNP proteins, that can act as splicing activators (enhancers) or repressors (silencers), respectively. Exonic SREs are called exonic splicing regulatory sequences (ESRseq) (Ke et al., 2011).


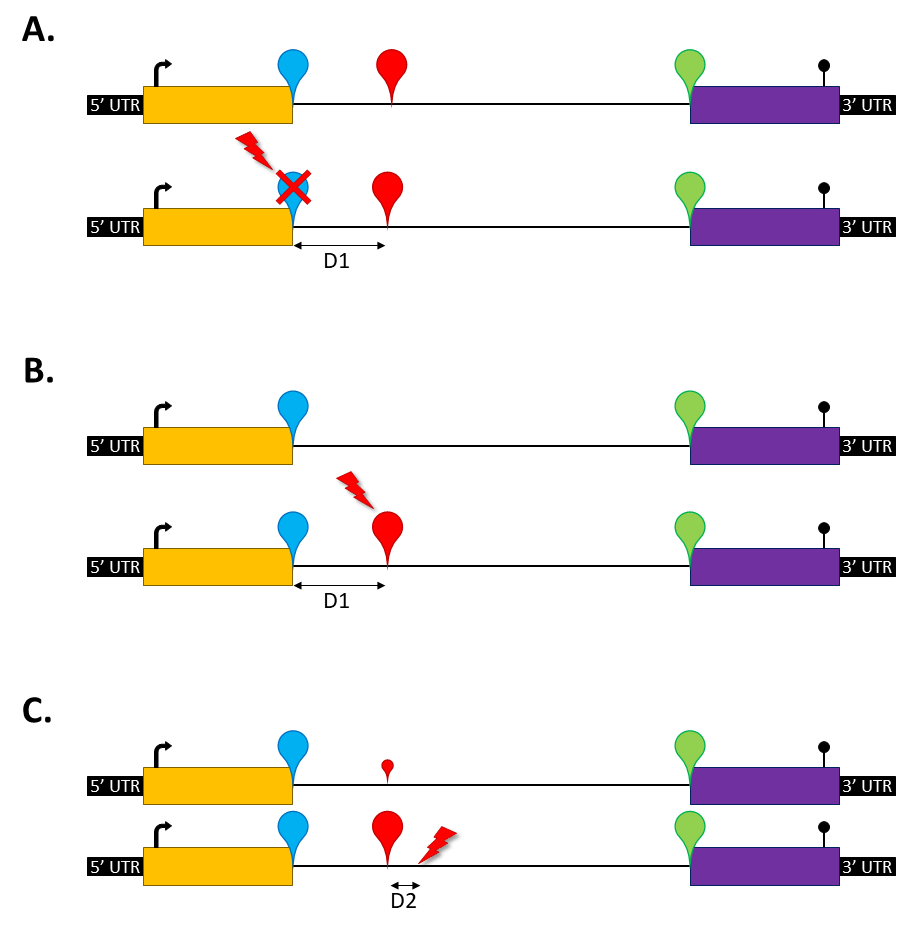


### **Supplementary** **Figure S3:** Mechanisms inducing the use of new splice site.

**A.** Shift of natural splice site: the variant alters natural splice site motif with the following of splice site is used in replacement. **B.** *De novo* splice site: the variant creates a new splicing motif leading to the use of the new splice site instead of the natural splice site. **C.** Cryptic splice site: the variant increases the capability of a pre-existing motif to be recognized by the spliceosome. UTR: UnTranslated Region, D1: distance between the natural splice site and the new splice site, D2: distance between the variant and the new splice site. Green drop: acceptor splice site, Blue drop: donor splice site, Red drop: new splice site.


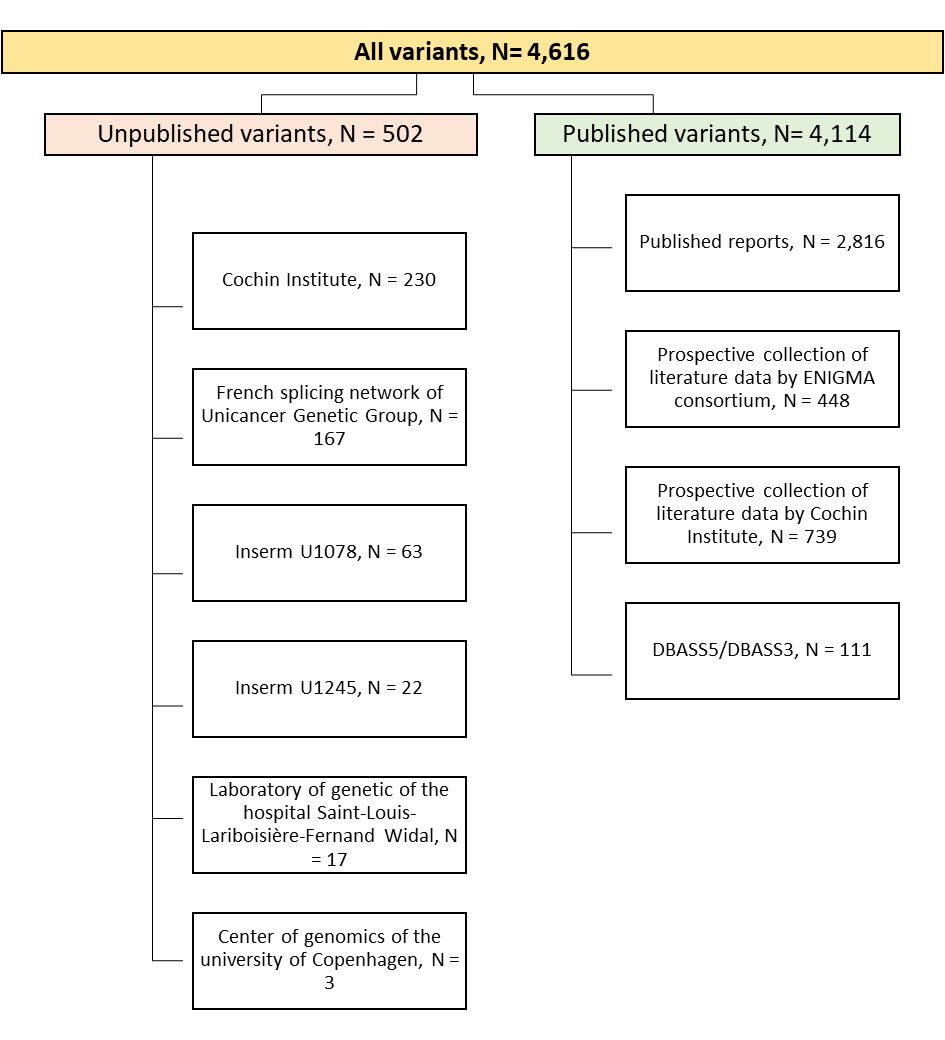


### **Supplementary** **Figure S4:** Collection of 4,616 variants with RNA *in vitro* studies.


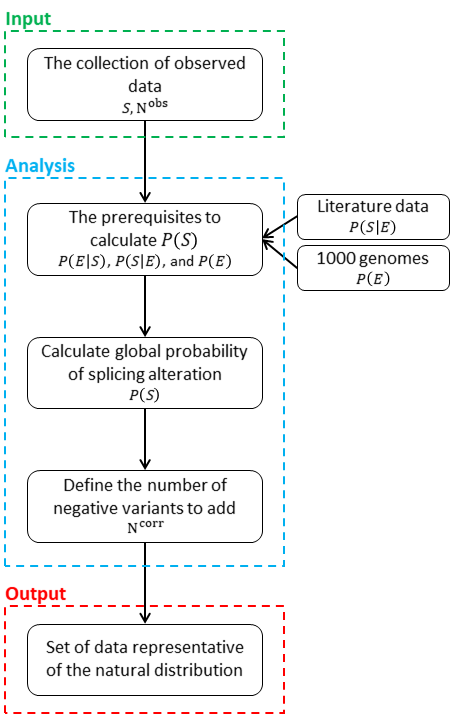


### **Supplementary Figure S5:** Workflow to get a set of data representative of the natural distribution of spliceogenic variants.


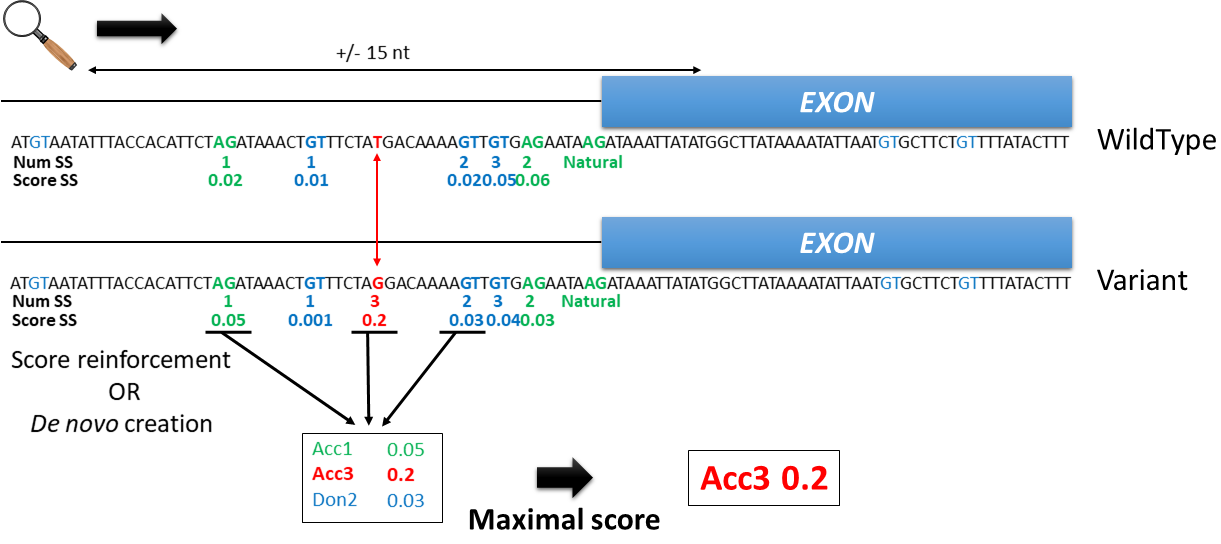


### **Supplementary Figure S6:** The strategy used by SPiP to detect cryptic splice activation.

In this example, the tool detects 2 AG signals and 3 GT signals in wild-type and variant sequences, plus a third *de novo* AG signal in the variant sequence, (*i.e.* potential splice sites). The tool compares the score of each potential splice site between wild-type and variant sequences, only the second donor site and the first acceptor sites have score reinforcement. For *de novo* acceptor site (Acc3) a score was obtained only on variant sequences. On these 3 remaining splice sites, the *de novo* splice site (Acc3) had the maximal score.


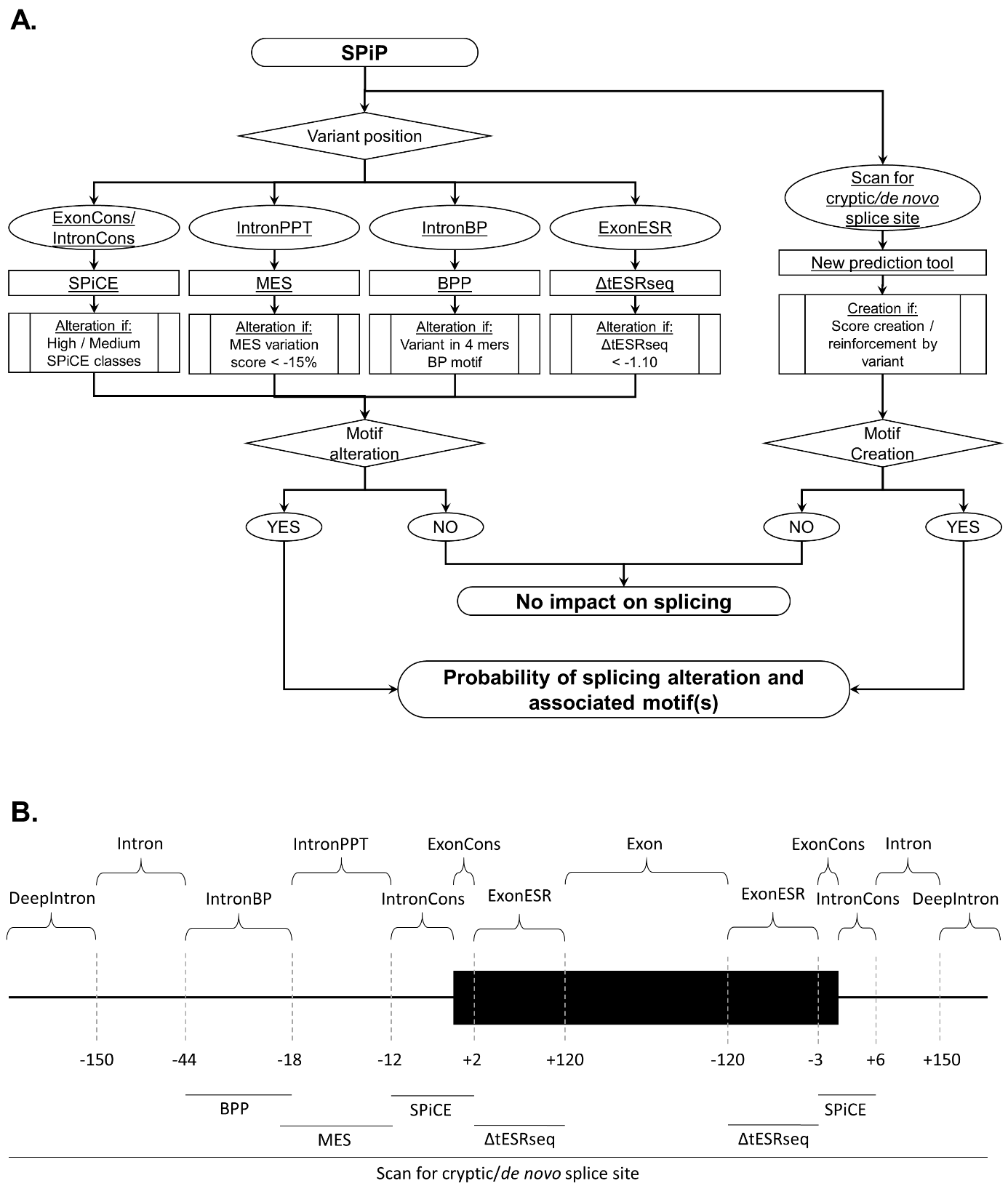


### **Supplementary Figure S7:** Pipeline of SPiP to detect which motif is probably impacted by a variant.

### **Supplementary** **Figure S8:** Distance between intronic variants and their nearest natural splice sites discriminates between variant-induced splice site shifts and pseudo-exons usage. The optimal threshold was a distance of 150 nt.


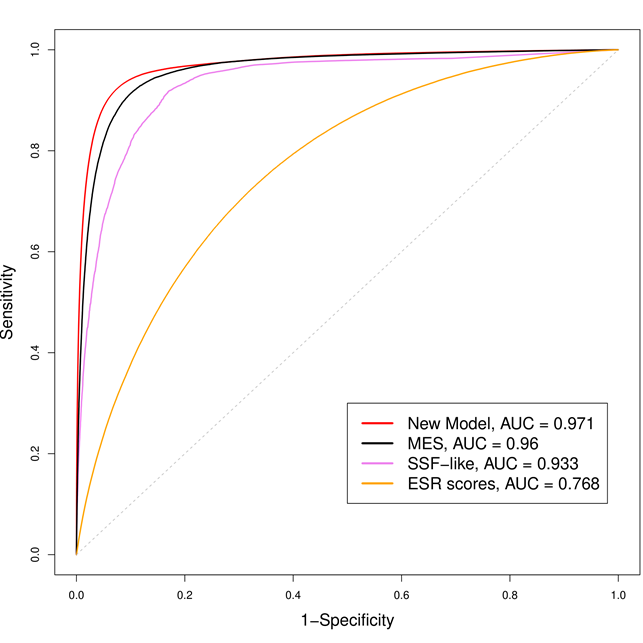


### **Supplementary Figure S9:** Prediction of splice site creation or reinforcement.

Performance of the new metascore model versus MaxEntScan (MES), Position Weight Matrix (SSF-like), Exonic Splicing Regulator scores (ESR). ROC curves were performed on the validation set of 71,137,680 splice sites

### **Supplementary Figure S10:** Comparison between SPiP, SpliceAI and SQUIRLS for the 100 iterations, n = 49,350 variants. AUC: Area Under the Curve; Se ; Sensitivity; Sp : Specificity


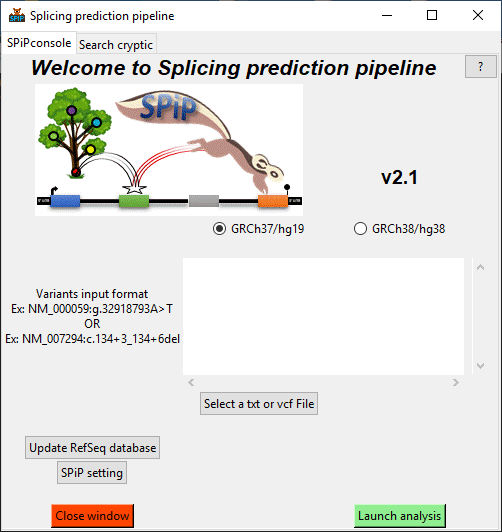


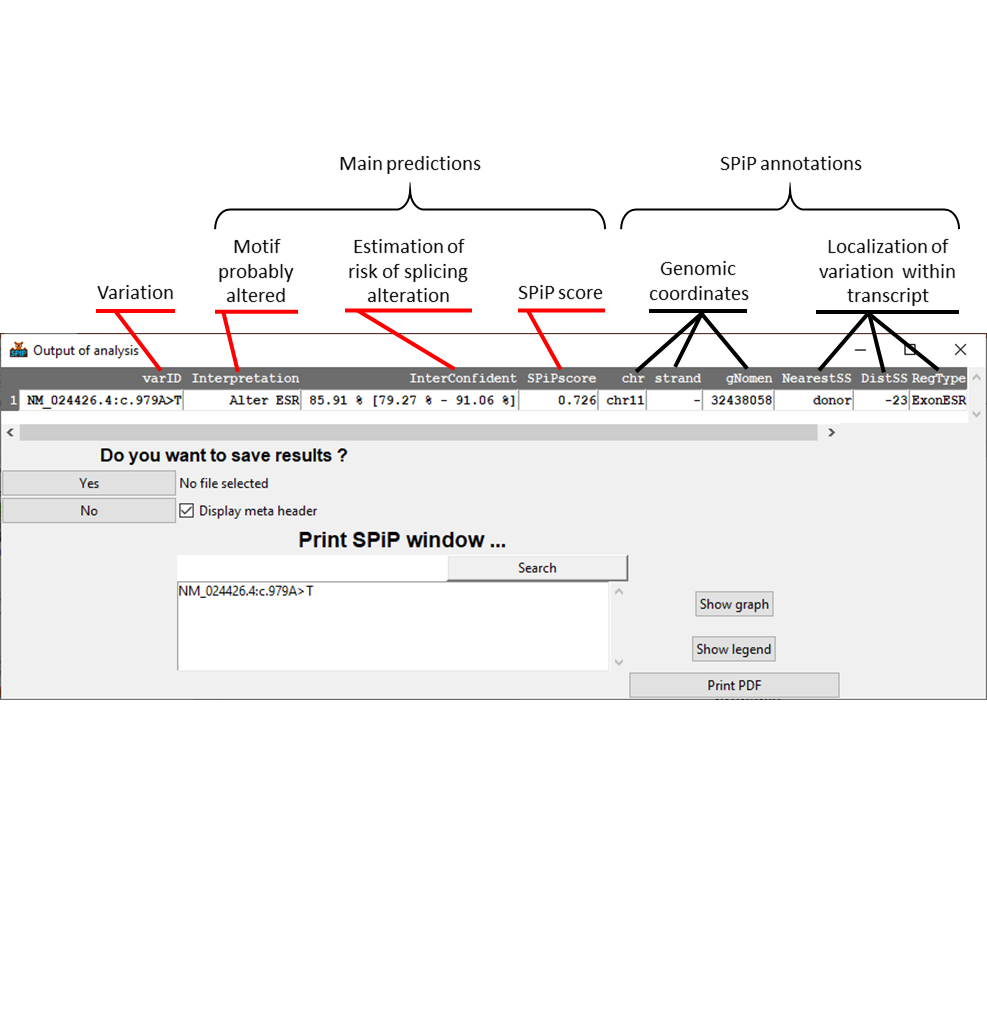


### **Supplementary Figure S11:** Screenshot of SPiP console for Windows version and an example of SPiP outputs.

## **REFERENCES**

Agresti A, Coull BA. 1998. Approximate is Better than “Exact” for Interval Estimation of Binomial Proportions. Am Stat 52:119–126.

Corvelo A, Hallegger M, Smith CWJ, Eyras E. 2010. Genome-Wide Association between Branch Point Properties and Alternative Splicing. PLoS Comput Biol 6:.

Desmet F-O, Hamroun D, Lalande M, Collod-Béroud G, Claustres M, Béroud C. 2009. Human Splicing Finder: an online bioinformatics tool to predict splicing signals. Nucleic Acids Res 37:e67–e67.

Erkelenz S, Theiss S, Otte M, Widera M, Peter JO, Schaal H. 2014. Genomic HEXploring allows landscaping of novel potential splicing regulatory elements. Nucleic Acids Res 42:10681–10697.

Giacomo DD, Gaildrat P, Abuli A, Abdat J, Frébourg T, Tosi M, Martins A. 2013. Functional Analysis of a Large set of BRCA2 exon 7 Variants Highlights the Predictive Value of Hexamer Scores in Detecting Alterations of Exonic Splicing Regulatory Elements. Hum Mutat 34:1547–1557.

Grodecká L, Buratti E, Freiberger T. 2017a. Mutations of Pre-mRNA Splicing Regulatory Elements: Are Predictions Moving Forward to Clinical Diagnostics? Int J Mol Sci 18:1668.

Grodecká L, Hujová P, Kramárek M, Kršjaková T, Kováčová T, Vondrášková K, Ravčuková B, Hrnčířová K, Souček P, Freiberger T. 2017b. Systematic analysis of splicing defects in selected primary immunodeficiencies-related genes. Clin Immunol 180:33–44.

Houdayer C, Caux‐Moncoutier V, Krieger S, Barrois M, Bonnet F, Bourdon V, Bronner M, Buisson M, Coulet F, Gaildrat P, Lefol C, Léone M, et al. 2012. Guidelines for splicing analysis in molecular diagnosis derived from a set of 327 combined in silico/in vitro studies on BRCA1 and BRCA2 variants. Hum Mutat 33:1228–1238.

Ke S, Shang S, Kalachikov SM, Morozova I, Yu L, Russo JJ, Ju J, Chasin LA. 2011. Quantitative evaluation of all hexamers as exonic splicing elements. Genome Res 21:1360–1374.

Leman R, Gaildrat P, Gac GL, Ka C, Fichou Y, Audrezet M-P, Caux-Moncoutier V, Caputo SM, Boutry-Kryza N, Léone M, Mazoyer S, Bonnet-Dorion F, et al. 2018. Novel diagnostic tool for prediction of variant spliceogenicity derived from a set of 395 combined in silico/in vitro studies: an international collaborative effort. Nucleic Acids Res 46:11656–11657.

Leman R, Tubeuf H, Raad S, Tournier I, Derambure C, Lanos R, Gaildrat P, Castelain G, Hauchard J, Killian A, Baert-Desurmont S, Legros A, et al. 2020. Assessment of branch point prediction tools to predict physiological branch points and their alteration by variants. BMC Genomics 21:86.

Lim KH, Ferraris L, Filloux ME, Raphael BJ, Fairbrother WG. 2011. Using positional distribution to identify splicing elements and predict pre-mRNA processing defects in human genes. Proc Natl Acad Sci 108:11093–11098.

Mercer TR, Clark MB, Andersen SB, Brunck ME, Haerty W, Crawford J, Taft RJ, Nielsen LK, Dinger ME, Mattick JS. 2015. Genome-wide discovery of human splicing branchpoints. Genome Res 25:290–303.

Mueller WF, Larsen LSZ, Garibaldi A, Hatfield GW, Hertel KJ. 2015. The Silent Sway of Splicing by Synonymous Substitutions. J Biol Chem 290:27700–27711.

Nazari I, Tayara H, Chong KT. 2019. Branch Point Selection in RNA Splicing Using Deep Learning. IEEE Access 7:1800–1807.

Paggi JM, Bejerano G. 2018. A sequence-based, deep learning model accurately predicts RNA splicing branchpoints. RNA 24:1647–1658.

Pertea M, Lin X, Salzberg SL. 2001. GeneSplicer: a new computational method for splice site prediction. Nucleic Acids Res 29:1185–1190.

Raponi M, Kralovicova J, Copson E, Divina P, Eccles D, Johnson P, Baralle D, Vorechovsky I. 2011. Prediction of single-nucleotide substitutions that result in exon skipping: identification of a splicing silencer in BRCA1 exon 6. Hum Mutat 32:436–444.

Reese MG, Eeckman FH, Genome H, Group I. 1995. Novel Neural Network Prediction Systems for Human Promoters and Splice Sites.

Shapiro MB, Senapathy P. 1987. RNA splice junctions of different classes of eukaryotes: sequence statistics and functional implications in gene expression. Nucleic Acids Res 15:7155–7174.

Signal B, Gloss BS, Dinger ME, Mercer TR. 2018. Machine learning annotation of human branchpoints. Bioinformatics 34:920–927.

Soemedi R, Cygan KJ, Rhine CL, Wang J, Bulacan C, Yang J, Bayrak-Toydemir P, McDonald J, Fairbrother WG. 2017. Pathogenic variants that alter protein code often disrupt splicing. Nat Genet 49:848–855.

Soukarieh O, Gaildrat P, Hamieh M, Drouet A, Baert-Desurmont S, Frebourg T, Tosi M, Martins A. 2016. Exonic splicing mutations are more prevalent than currently estimated and can be predicted by using In Silico tools. PLoS Genet 12:.

Sterne-Weiler T, Howard J, Mort M, Cooper DN, Sanford JR. 2011. Loss of exon identity is a common mechanism of human inherited disease. Genome Res 21:1563–1571.

Teraoka SN, Telatar M, Becker-Catania S, Liang T, Önengüt S, Tolun A, Chessa L, Sanal Ö, Bernatowska E, Gatti RA, Concannon P. 1999. Splicing Defects in the Ataxia-Telangiectasia Gene, ATM: Underlying Mutations and Consequences. Am J Hum Genet 64:1617–1631.

The 1000 Genomes Project Consortium. 2015. A global reference for human genetic variation. Nature 526:68–74.

Tournier I, Vezain M, Martins A, Charbonnier F, Baert‐Desurmont S, Olschwang S, Wang Q, Buisine MP, Soret J, Tazi J, Frébourg T, Tosi M. 2008. A large fraction of unclassified variants of the mismatch repair genes MLH1 and MSH2 is associated with splicing defects. Hum Mutat 29:1412–1424.

Xiong HY, Alipanahi B, Lee LJ, Bretschneider H, Merico D, Yuen RKC, Hua Y, Gueroussov S, Najafabadi HS, Hughes TR, Morris Q, Barash Y, et al. 2015. The human splicing code reveals new insights into the genetic determinants of disease. Science 347:.

Yeo G, Burge CB. 2004. Maximum Entropy Modeling of Short Sequence Motifs with Applications to RNA Splicing Signals. J Comput Biol 11:377–394.

Zhang Q, Fan X, Wang Y, Sun M, Shao J, Guo D. 2017. BPP: a sequence-based algorithm for branch point prediction. Bioinformatics 33:3166–3172.
